# Experimental and numerical analysis of pGFRP and wood cross-arm in latticed tower: a comprehensive study of mechanical deformation and flexural creep

**DOI:** 10.1038/s41598-024-83634-7

**Published:** 2025-01-09

**Authors:** Amir Abd Latif, Mohamad Ridzwan Ishak, Muhammad Rizal Razman, Noorfaizal Yidris, Mohamed Yusoff Mohd Zuhri, Muhammad Asyraf Muhammad Rizal, Zuliskandar Ramli

**Affiliations:** 1 Department of Aerospace Engineering, University Putra Malaysia (UPM), 43400 Serdang, Selangor Malaysia; 2 Aerospace Malaysia Research Centre (AMRC), University Putra Malaysia (UPM), 43400 Serdang, Selangor Malaysia; 3 Laboratory of Biocomposite Technology, Institute of Tropical Forestry and Forest Products (INTROP), University Putra Malaysia (UPM), 43400 Serdang, Selangor Malaysia; 4 Research Centre for Sustainability Science and Governance (SGK), Institute for Environment and Development (LESTARI), Universiti Kebangsaan Malaysia (UKM), 43600 Bangi, Selangor Malaysia; 5 Research Centre for Advanced Engineering Materials and Composites (AEMC), Department of Mechanical and Manufacturing Engineering, University Putra Malaysia (UPM), 43400 Serdang, Selangor Malaysia; 6 Engineering Design Research Group (EDRG), Faculty of Mechanical Engineering, Universiti Teknologi Malaysia, 81310 Johor Bahru, Johor Malaysia; 7 Centre for Advanced Composite Materials (CACM), Universiti Teknologi Malaysia, 81310 Johor Bahru, Johor Malaysia; 8 Institute of the Malay World and Civilisation (ATMA), Universiti Kebangsaan Malaysia (UKM), 43600 Bangi, Selangor Malaysia

**Keywords:** pGFRP composites, Cross-arm, Wood timber, Transmission tower, Flexural properties, Load-deflection, Materials science, Engineering, Mechanical engineering

## Abstract

The adoption of pultruded glass fibre-reinforced polymer (pGFRP) composites as a substitute for traditional wooden cross-arms in high transmission towers represents a relatively novel approach. These materials were selected for their high strength-to-weight ratio and lightweight properties. Despite various studies focusing on structures improvement, there still have a significant gap in understanding the deformation characteristics of full-scale cross-arms under actual operational loads. Existing study often concentrate on small coupon scale and laboratory condition, leaving a gap in understanding how the cross-arm behavior in full-scale acting on actual weather condition. This study aims to investigate the load-deflection and long-term creep behavior of a pGFRP cross-arm installed in a 132 kV transmission tower. The pGFRP cross-arm was load-tested on a customized rig in an open environment. Using the cantilever beam concept, deflection was analyzed and compared to wood cross-arms. Finite element analysis validated results, and long-term deformation under high-stress loads was assessed. The pGFRP cross-arms showed lower deflection at working loads compared to Balau wood, due to the latter’s higher elastic modulus and flexibility specifically at Point Y3, the critical issues necessitated reinforcement strategies. pGFRP cross-arms withstood higher bending stress, showing 32% less deflection under normal conditions and 15% less under broken wire conditions than Balau wood. Additionally, the creep strength of wood was 34% lower than that of pGFRP cross-arms. Besides that, the pGFRP cross-arm have highest elastic modulus than Balau-wood, shows that the composite cross-arm have better structural strength, resisting deformation and higher flexibility materials. Finite element analysis (FEA) confirmed these results with the relative error between them less than 1%. Consequently, the investigation into pGFRP cross-arm deformation behavior in this paper serves as a foundational framework for future research endeavors specifically for high transmission tower and other structural application.

## Introduction

Transmission towers used wooden cross-arms when power transmission lines were initially installed in Malaysia^1^. The usage of Chengal wood (Neobalanocarpus) for cross-arms in 66 kV towers began in 1929 and continued into the 1960s when it was used in 132 kV suspension towers^2^. Approximately 22 different types of wood were used for the cross-arm fabrication process in high transmission towers during this period. Wood became highly demand in structure fabrication due to its advantages, including low production cost, abundant cheapest resources and easy availability^3^.

Balau wood and pultruded glass fiber reinforced polymer (pGFRP) composites are gaining attention for their use in cross-arm structures of latticed transmission towers due to their distinct flexural properties. Balau wood, known for its exceptional bending strength and modulus, outperforms other materials like Schizolobium amazonicum Herb, Merbau, and laminated veneer lumber (LVL)^4–6^. These qualities make it ideal for transmission towers exposed to varying environmental conditions and mechanical loads. Similarly, pGFRP composites present a lightweight and high-strength option with outstanding resistance to environmental degradation, making them well-suited for long-term use in harsh outdoor environments^7^. Comparative studies highlight the structural robustness of Balau wood alongside the adaptability and durability of pGFRP composites. These findings demonstrate the potential of both materials to improve the performance and longevity of cross-arm structures in transmission towers, meeting demands for high mechanical strength and environmental resilience^8^. Besides that, some researchers have proved the used of hybrid-reinforced wooden beam with environmental friendly wooden beams can increased load bearing capacity by over 50% and prevent failure like crack development and maintain aesthetics^9^. Therefore, understanding the flexural properties of Balau wood and pGFRP composites is crucial for developing advanced, reliable cross-arm solutions in latticed transmission towers.

The shift from wooden cross-arms to pultruded glass fiber reinforced polymer (pGFRP) composites has been motivated by the limitations of wood, which, due to aging and natural defects, often fails to achieve a service life beyond 14 years^10–12^. Introduced in Malaysia in 1999, pGFRP composites provide lightweight and high-strength alternatives, offering enhanced durability in transmission tower applications^13–16^. Their advantages include superior creep resistance and stability in harsh conditions, design flexibility through the pultrusion process for mass production, and extended service life enabled by innovations such as honeycomb sandwich structures^17–19^. In contrast, Balau wood, historically valued for its natural strength and resistance to environmental factors, has been found to perform well in flexural modulus but lags behind pGFRP composites in creep resistance. Despite the clear benefits of pGFRP, concerns about its long-term performance under operational conditions remain, emphasizing the need for continued research to optimize its role in energy transmission systems. Table 1 shows the recent development and advancement of geopolymer applications such as pGFRP composite and balau woods products.

**Table 1:** Recents application of pGFRP composite and balau woods products.

Types of material	Application	Product	Ref.
Pultruded glass fiber reinforced polymer composite	Bridge engineering	Bridge decks, railing and structural members	^20,21^
		Bridge cables	^22^
	Structural reinforcements	Beam and pin-bearing	^23–25^
	Building components	Roofs, plate, shell elements and linear elements	^26^
	Emergency applications	Shelter after earthquakes, flood and all disastrous	^27–29^
	Energy sectors	Transmission tower	^30^
	Rehabilitation and retrofitting	Foundation for machines	^31^
	Aerospace applications	Drilling compartment, space launches and satellites	^32,33^
	Automotive compartment	Chassis and bumper beam	^34,35^
Balau woods	Economical structural	Timber design	^36^
	Army field	Shooting target surface	^37^
	Water transportation	Wooden ship	^38^
	Heat treatment	Wood flour	^39^
	Energy sectors	Transmission tower	^40^

Although pGFRP cross-arm members are now widely used in suspension transmission towers, they are subjected to complex failure mechanisms due to multi-axial loading. These cross-arm structures are constantly exposed to tropical climates, wind turbulence, and mechanical loading from insulators and cables throughout their service life, leading to degradation^12,15,16^. Despite these advantages, their performance has been observed to decline over time due to various environmental factors. Studies have shown that tropical climates, wind turbulence, mechanical loading from insulators and cables, and factors such as creep, temperature fluctuations, and excessive humidity can accelerate the degradation rate of pGFRP composite cross-arms^41,42^. Comparative research on the flexural creep behavior of pGFRP composites has highlighted the impact of stacking sequence on long-term performance, further emphasizing the need for optimized designs to enhance durability^43^. Additionally, the exploration of green composites and alternative materials presents potential avenues for improving the service life of cross-arm structures in transmission towers^44^. Understanding these degradation mechanisms and material behaviors is crucial for developing more resilient and long-lasting composite cross-arms.

The properties of composites, such as pGFRP, are significantly influenced by factors like the type of polymer, fiber content, orientation, and treatment^45–47^. These factors play a crucial role in determining the mechanical properties of the composite, including stiffness, flexural characteristics, energy absorption, and load-carrying capacity^45^. Understanding the behavior of these composites under specific loads is essential for designing and ensuring the structural integrity of components like cross-arm assemblies^42,48,49^. Research has shown that the deflection behavior of structural members under various loads has been extensively studied, providing valuable insights into the design and performance of such structures^50–52^. For instance, the use of pGFRP as replacements for traditional materials in transmission towers has been a subject of recent investigation^50,53^. Studies have highlighted the benefits of composite cross-arms, such as excellent insulation properties, high mechanical strength, lightweight nature, corrosion resistance, and ease of installation^54^. Other researchers have investigated the GFRP composite can significantly enhance the performance and durability of construction practices by strengthening innovative configuration and imrpoved ultimate load capacity for almost 15%^55^.

In a worst-case scenario, the failure of tower line assemblies due to inadequate strength and damping properties of pGFRP cross-arm assemblies could lead to catastrophic collapses^56^. While existing research has primarily focused on the behavior of coupon-scale pGFRPs, there is a need for more in-depth studies on the actual-scale behavior of cross-arms^57^. Understanding the mechanical properties, damping capacities, and energy absorption efficiencies of these composites is crucial for ensuring the long-term functionality and safety of transmission structures. In conclusion, the properties of composites like pGFRP are multifaceted and depend on various factors. Research on the mechanical behavior, strength, and damping properties of these materials is essential for the design and reliability of structural components like cross-arms in transmission towers. Further studies focusing on the actual-scale behavior of these composites are warranted to enhance our understanding of their performance in real-world applications.

Numerous researchers have explored both experimentally and theoretically the deflection patterns of structural elements, particularly focusing on initially straight cantilever beams under specific loads. Singhal et al.^58^ have proposed a theoretical framework to analyze both small and large deflections in cantilever beams. In the context of small deflection theory, the vertical or transverse deflections at the cantilever tip exhibit linearity, while longitudinal or horizontal deflections are negligible. Conversely, large deflection analysis accounts for realistic, nonlinear transverse, and longitudinal deflections. Despite this, both small and large deflection approaches produce similar outcomes within the linear elastic range, suggesting the adoption of the simpler small deflection method for engineering design purposes^59,60^. Furthermore, analyzing small deflection behavior is crucial for establishing fundamental structural design requirements and determining the structural applicability range. Analyzing small deflection behavior in structural elements, such as cantilever beams, is crucial for several reasons. Firstly, studying small deflections allows engineers to establish fundamental structural design requirements by providing insights into the linear behavior of the structure under load. By understanding how the beam deforms within the small deflection range, engineers can determine the stiffness of the structure and predict its response to various loads accurately. Secondly, analyzing small deflections helps in determining the structural applicability range of the design. By studying the linear behavior of the beam, engineers can identify the limits within which the structure will perform as intended. This information is essential for ensuring that the design meets safety standards, functional requirements, and performance expectations. In summary, analyzing small deflection behavior in cantilever beams and other structural elements is essential for establishing the basic design requirements, determining the structural applicability range, and providing a reliable basis for engineering design decisions. The progress of composite and non-composite beam studies across various types of beams is summarized in Table 2, showcasing the ongoing research in this area conducted by different researchers.

**Table 2:** Issues concerning cross-arm structures and the suggestions provided by diverse research endeavors.

Research	Type of Beam	Finding	Ref.
Experimental and theoretical study on small and large deflection due to concentrated tip load	Single composite cantilever beam	The study used finite element analysis solution to verify a novel method for calculating large deflection by utilising the fundamental idea of small deflection.	^58–60^
Bending behaviour study of a beam subjected to combined tip loading	Single cantilever beam	The deflection characteristic equation of the beam for any combination of loading and deflection parameters was introduced in this study.	^61–64^
Mechanical behaviour of a beam by using Numerical analysis study	4 members composite assembled beam	The experiment from the finite element analysis was used to validate the composite cross-arm’s maximum deflection behaviour under simultaneous load.	^65,66^
Bending behaviour study of a beam subjected to different wire loading condition	3 members of wood assembled beam	Maximum deflection behaviour of full-scale wooden cross-arm was investigated experimentally and numerically.	^40^
Experimental and Numerical analysis study of Creep behaviour	3 members of composite assembled beam	For long-term applications, the bracing system is used for the cross-arm structures and suggested as a way to increase service life and thereby decrease maintenance work.	^48^

Regrettably, the current data and information are inflated, straying from the actual mechanical performance of pGFRP cross-arms. Furthermore, many researchers have encountered challenges in proposing enhancements to cross-arm designs^67–69^. Munusamy et al.^65^ have study the mechanical propeties of composite cross-arm consist of pultruded rods and crimped metallic end-clamps. They supported the integration of these cross-arms with an FRP tower structure to reduce the horizontal phase spacing, thereby enabling the future compact transmission lines. Hussein et al.^40^, presented a conceptual design that install bracing system on cross-arm members in order to enhance the long term performance of the cross-arm. Meanwhile, Selvaraj et al.^70^ conducted studies on utilizing composite pultruded sections as substitutes for rolled steel angle sections in the X-braced panel design of transmission line towers, focusing on linear-elastic responses and considerations for buckling.

This study investigates the load deflection behavior of actual-size pGFRP cross-arms in a 132 kV transmission tower compared to traditional Balau wood cross-arms. The research utilizes the cantilever beam concept under normal and broken loading wire conditions. Results are compared with Finite Element Analysis (FEA) outcomes. The study aims to enhance the structural design of composite cross-arms for improved mechanical performance in future applications.

## Methodology

**Fig. 1:**
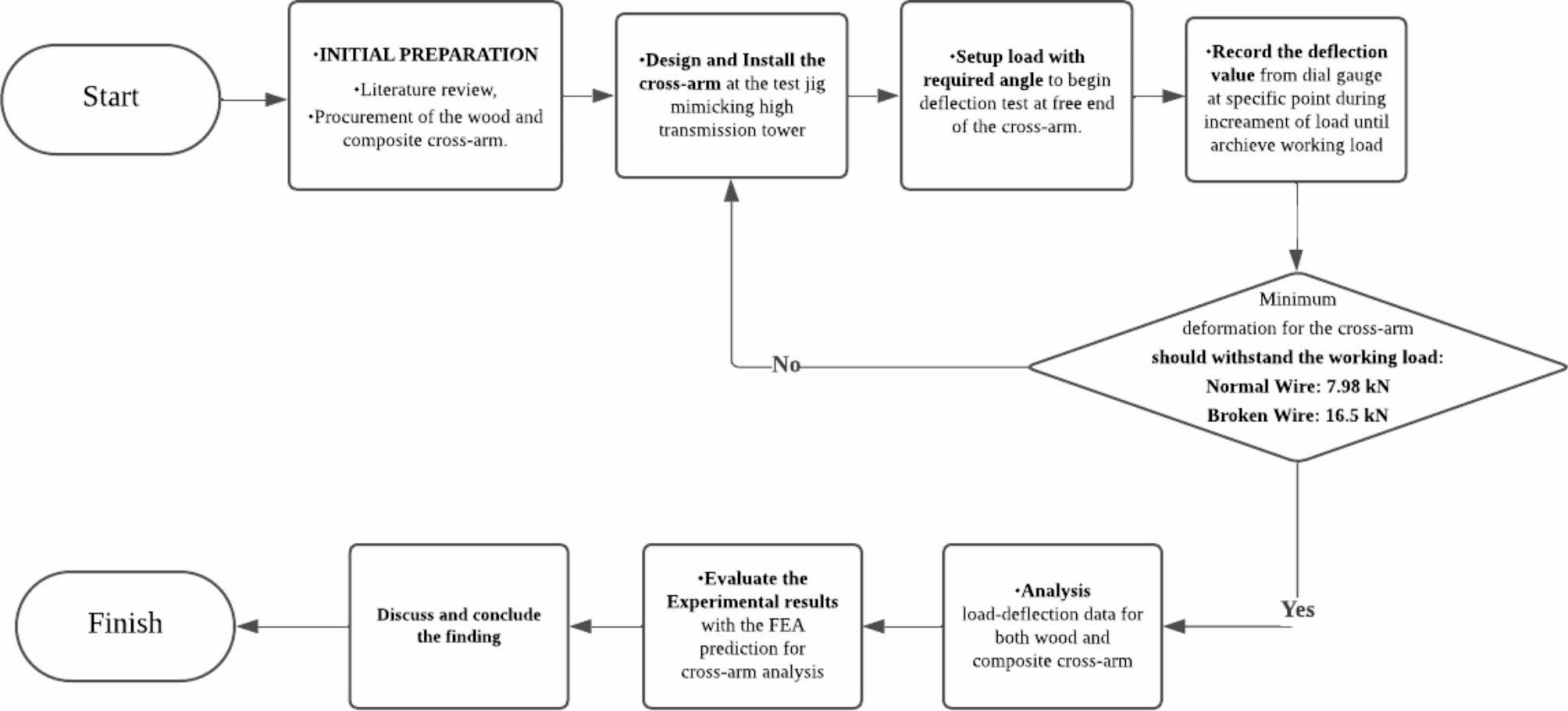
Flow chart of overall experimental setup.

The methodology for the research on wood and composite cross-arms involves a systematic approach starting with an extensive literature review and the procurement of Balau wood and pultruded Glass Fiber-Reinforced Polymer (pGFRP) composites cross-arms. The experimental setup involves installing the cross-arms on a test jig designed to replicate the structure of a high transmission tower. A deflection test is conducted by applying incremental loads at specific angles to the free end of the cross-arm, with deflection values recorded at each load increment using a dial gauge until the working load is achieved. The minimum deformation for the cross-arm should withstand both normal wire (7.98 kN) and broken wire (16.5 kN) loads as discuss in Sections "Working load for normal wire condition" and "Working load for broken wire condition". The collected data is tabulated, and a load-deflection graph is plotted. The results for the pGFRP cross-arm are compared to those of the Balau wood cross-arm to evaluate performance. Additionally, the experimental results for the pGFRP cross-arm are validated through Finite Element Analysis (FEA) predictions. Further elaboration on the materials and methods used in the research is provided in the following section. The comprehensive research methodology flowchart is depicted in Fig. 1.

### Materials

**Fig. 2:**
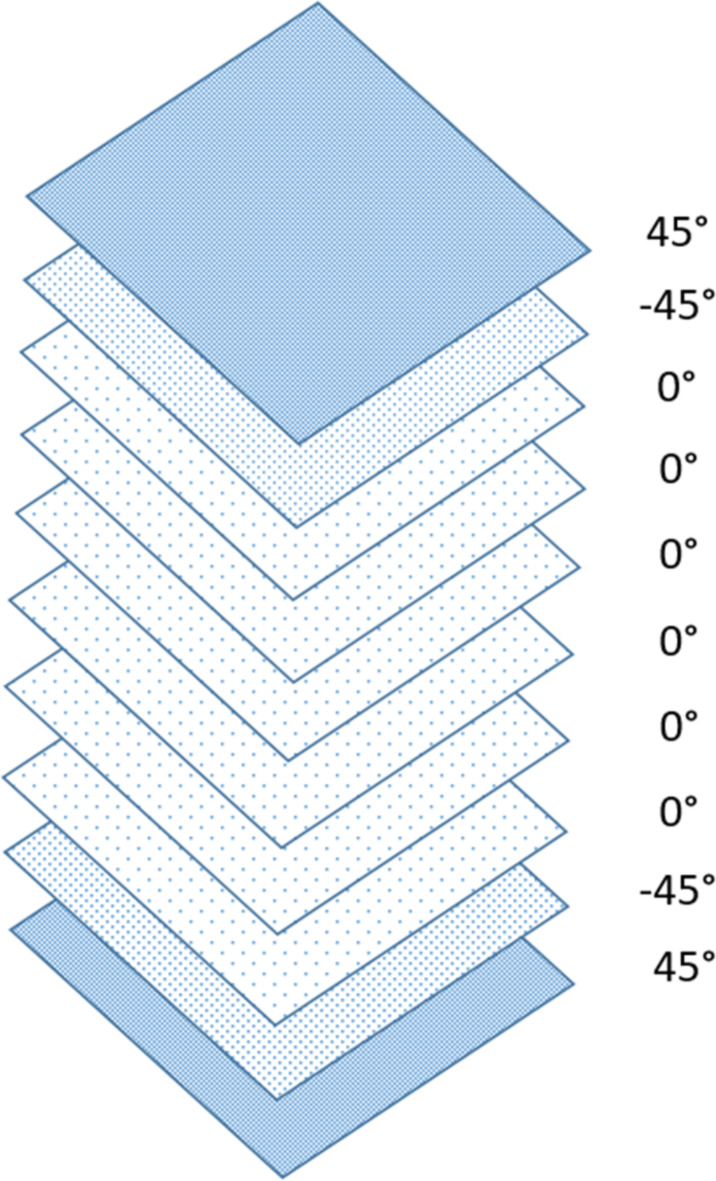
Composite stacking sequence of pultruded composite cross-arms.

This study examines pultruded glass fiber-reinforced polymer (pGFRP) cross-arms utilized in a 132 kV transmission tower in Malaysia, supplied by Electrius Sdn Bhd. These cross-arms, composed of one tie and two main members, are fabricated using the pultrusion process with E-glass fiber reinforcement and unsaturated polyester resin. The primary focus is on the main cross-arm member, which plays a critical role in supporting the transmission lines. The cross-arm comprises approximately 37% E-glass fiber and 63% unsaturated polyester resin. Table 3 provides detailed characteristics of the cross-arm coupon sample, while Fig. 2 illustrates the ten-layer pultruded composite structure with varying stacking sequences. However, the limitation of this research to highlights the challenge of developing sustainable raw materials, such as those incorporating alkaline activation, to minimize the environmental impact of such structures was not considered. Geopolymer production involves blending an alkaline activator with aluminosilicate-rich raw materials to form a complex aluminosilicate gel^71,72^. Factors like binder type, casting method, curing conditions and temperature, and the viscosity of the alkaline silicate significantly influence the mechanical properties of the geopolymer^73^.

**Table 3:** Coupon scale properties of pGFRP and Balau wood cross-arm^74,75^.

Properties	pGFRP	Balau-wood
Density	2580 kg/m^3^ – E-glass 1350 kg/m^3^ – Unsaturated polyester	850–1155 kg/m3
Texture	Fine, homogenously and unidirectional fibre along the matrix	Fine and even with deeply interlocked grain
Shrinkage	Low	High
Natural Durability	Low	Very High
Modulus of Elasticity	29.8 GPa	20.1 GPa
Modulus of Rupture	858.0 MPa	142.0 MPa

**Fig. 3:**
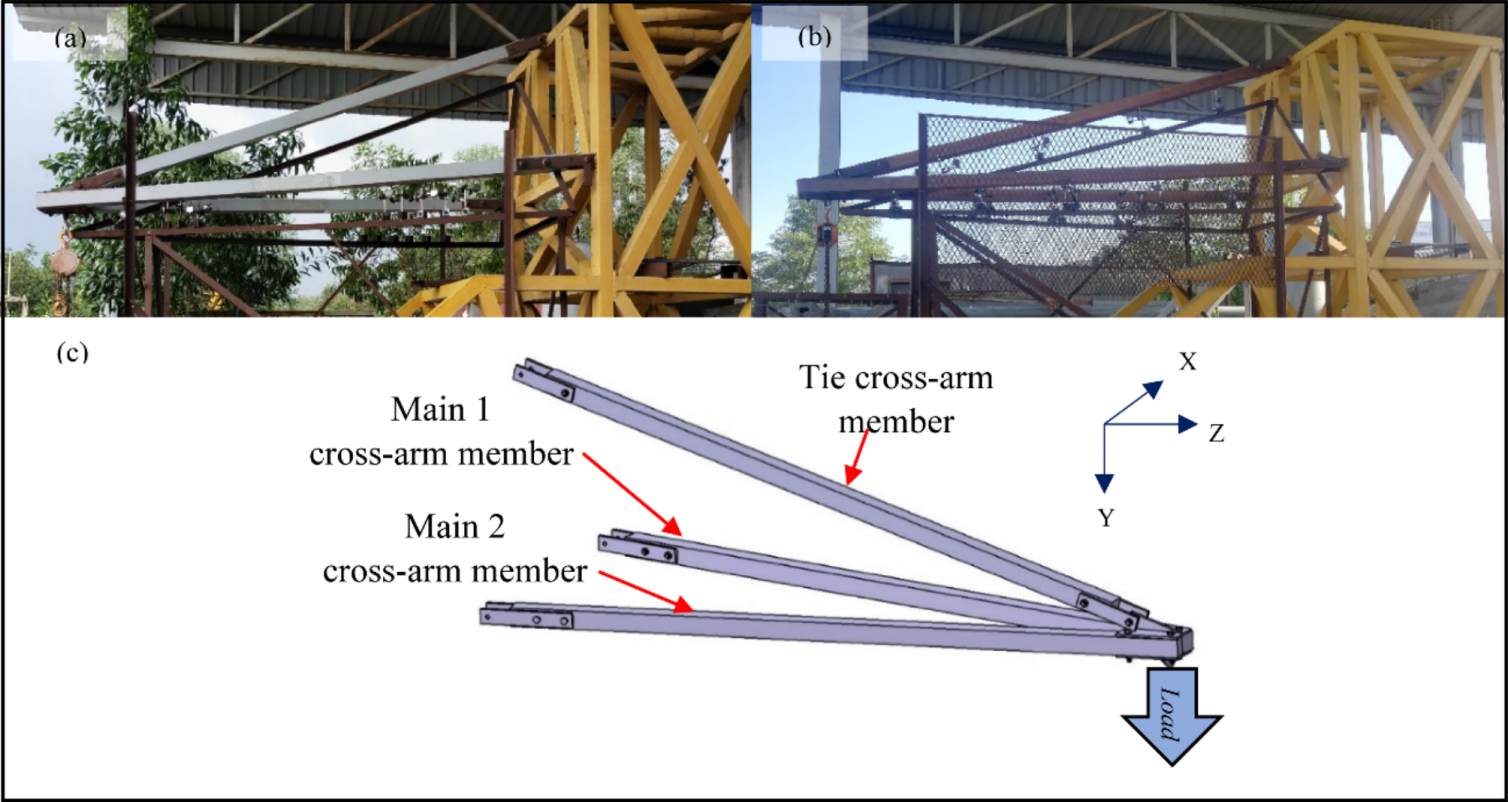
Complete assembly cross-arm members (**a**) Composite cross-arm, (**b**) Wood cross-arm (**c**) Isometric view.

The square part of both composite and wood cross-arm have same dimensions of 102 × 102 mm^2^ and shows a consistent, meeting the criteria set by the Tenaga Nasional Berhad (TNB) standard as shown in Fig. 3. The main members of the cross-arm have a length of 3,651 mm while the tie member was 3,472 mm length. Each cross-arm component was securely attached to the testing apparatus using nuts, bolts and fastening brackets. A load, adhering to the TNB standard workload for real-world conditions, was then applied to the open end of the cross-arm.

### Experimental setup for deformation test

**Fig. 4:**
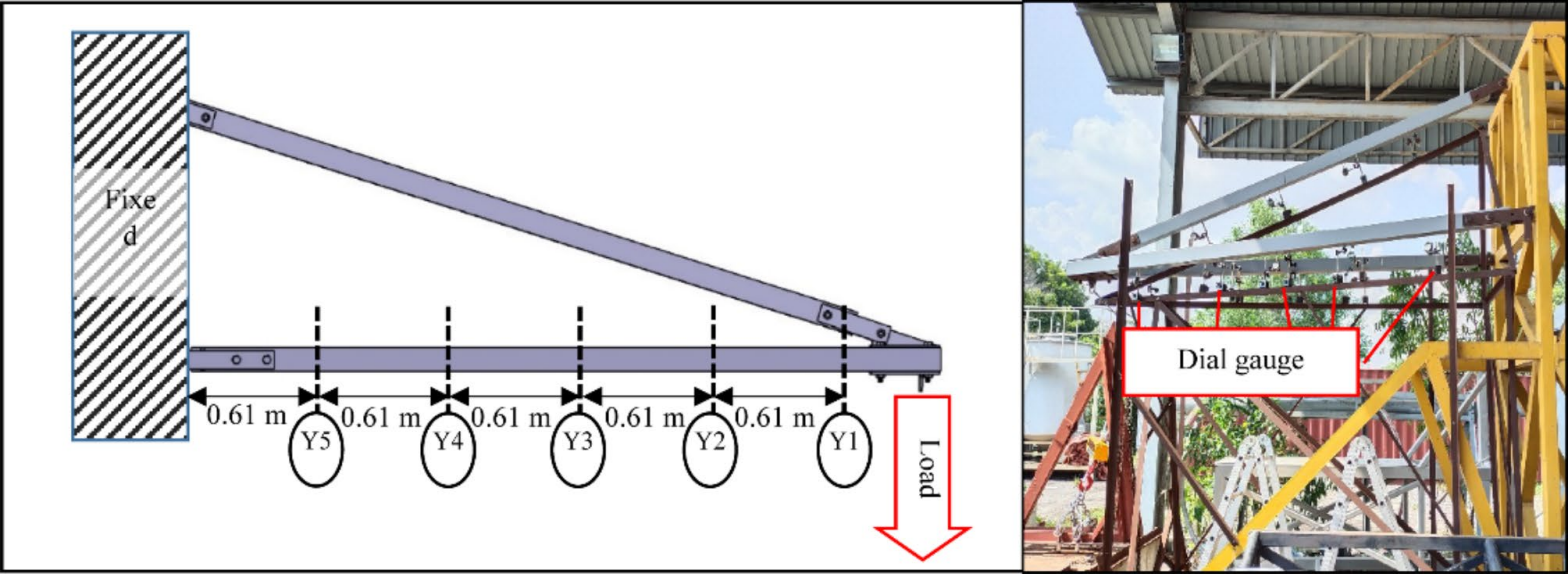
Illustration and actual positioning of dial gauges at intervals along the cross-arm during testing conditions.

Since the cross-arm operates similarly to a cantilever beam, its characteristics were evaluated through a two-point bending test. The testing approach was modified from research conducted by Asyraf et al. and Hussein et al.^23,76^ Dial gauges were placed at five specific points (Y1 to Y5) along each main member of the cross-arm to measure deflection, spaced 0.61 m apart, with Y1 positioned nearest to the free end as illustrated in Fig. 4. A 3-ton crane scale was utilized to gradually apply load at the free end until reaching the actual operational load specified by the TNB standard. This study evaluated two wire conditions including normal wire and broken wire.

Deflection behavior in the Y-axis direction of the cross-arm was observed under both normal and broken wire conditions. Further elaboration on the prescribed actual working loads for normal and broken wire conditions, as per TNB standards, is provided in sections “Working load for normal wire condition” and “Working load for broken wire condition”. The experiment was conducted outdoors to subject the cross-arm to real tropical weather conditions. Following the experiment, the minimum and maximum deflection values of the pGFRP cross-arm were compared to those of the Balau wood cross-arm investigated by Hussein et al.^76^ The cantilever beam principle was also incorporated into the Finite Element Analysis (FEA) model, with experimental results utilized for validating numerical predictions.

#### Working load for normal wire condition

**Fig. 5:**
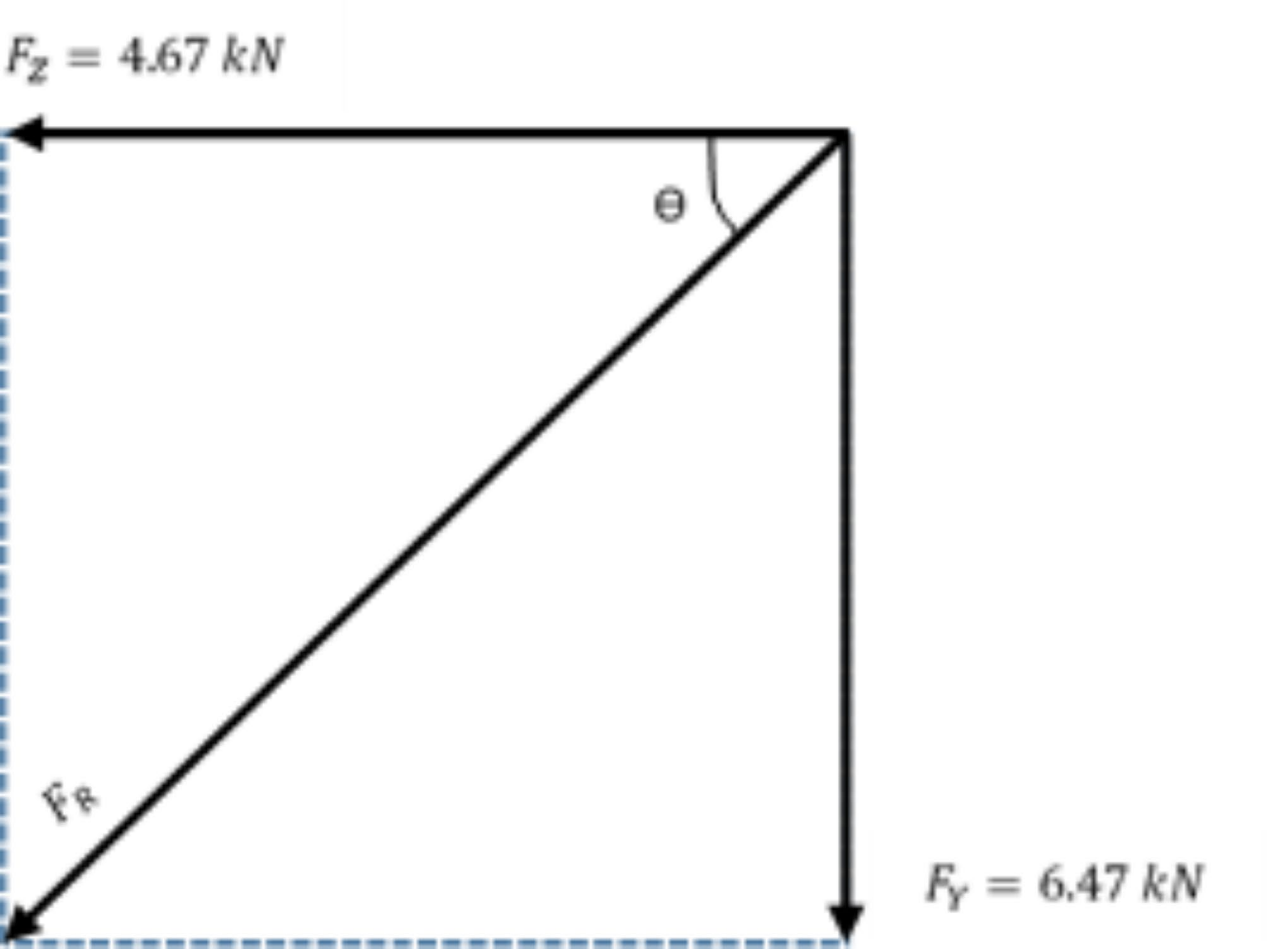
Force projection of working load in normal wire condition.

In TNB specification, the standard normal wire condition refer to the force acting in vertical and transverse direction for the transmission conductor wire connected to the cross-arm properly^76^. The working load is a resultant force of 132 kV 13 L pGFRP cross-arm in transverse and vertical directions which are 4.67 kN and 6.47 kN respectively as shown in Fig. 5.

By applying Pythagoras’ Theorem, the resultant force (F_R_) obtained from both transverse and vertical force are as below:

$$\:{F}_{R}=\sqrt{{{F}_{Y}}^{2}+{{F}_{Z}}^{2}}$$

$$\:{F}_{R}=\sqrt{{4.67}^{2}+{6.47}^{2}}$$

$$\:{F}_{R}=7.98\:kN$$

Afterwards, the angle of the resultant force is define as:

$$\:\theta\:={tan}^{-1}\left(\frac{{F}_{Y}}{{F}_{Z}}\right)$$

$$\:\theta\:={tan}^{-1}\left(\frac{6.47}{4.67}\right)$$

$$\:\theta\:=54.2^\circ\:$$

By using the 1.0 safety factor, the working load was applied to the cross-arm at an angle of 54.2° from the horizontal plane. The applied load was increased by steps of 1000 N until the working load of 7.98 kN for normal wire condition was reached. Then the obtained deflection value was recorded.

#### Working load for broken wire condition

**Fig. 6:**
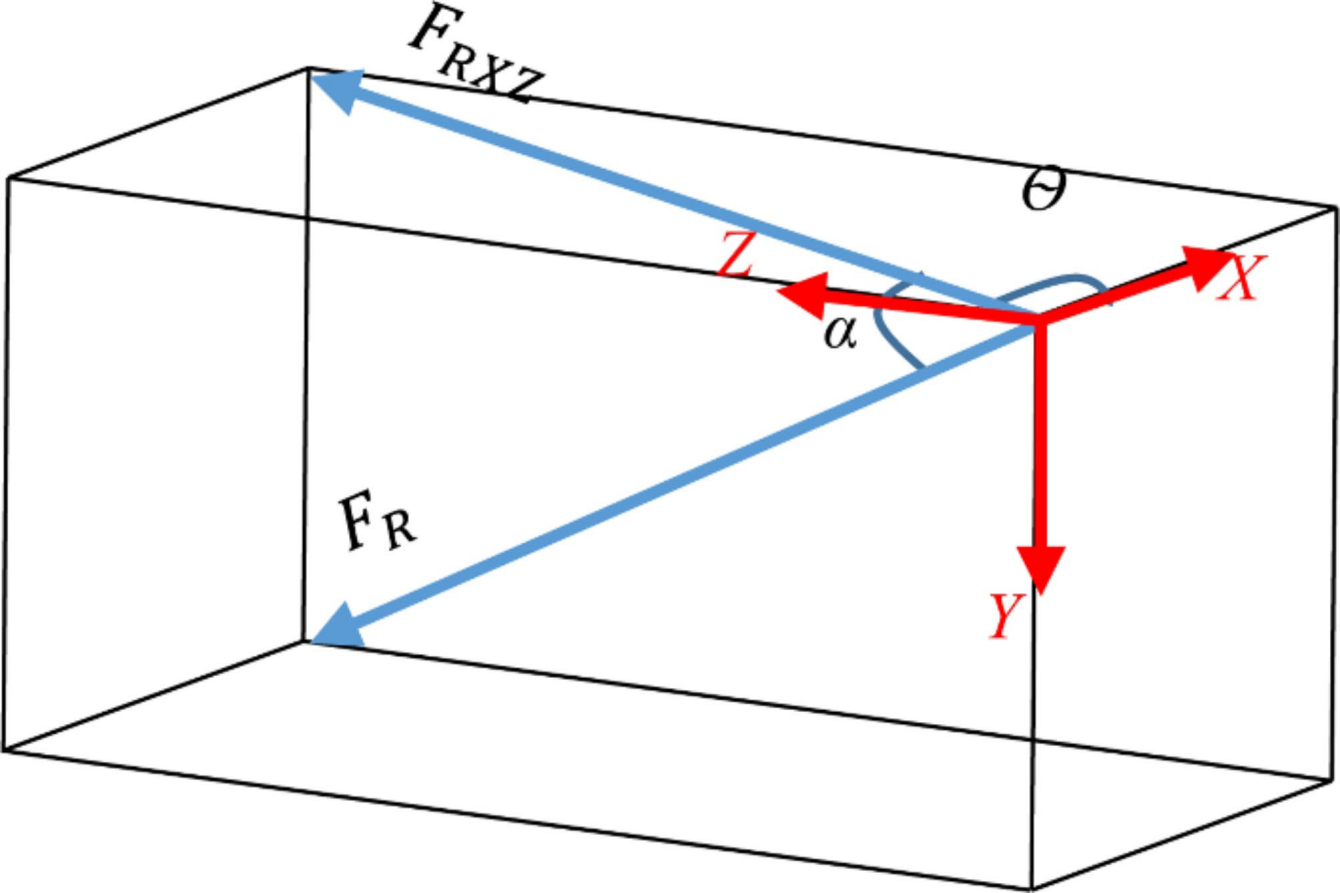
Force projection of working load in normal wire condition.

The broken wire condition in TNB specification refer to the force acting in vertical, transverse and longitudinal directions for the broken transmission conductor wire^76^. The working load is a resultant force of 132 kV 13 L pGFRP cross-arm in vertical, transverse and longitudinal directions where the values are 3.43 kN, 4.97 kN and 15.35 kN respectively as shown in Fig. 6.

By applying Pythagoras’ Theorem, the resultant force (F_R_) obtained from vertical, transverse and longitudinal forces are as below:

$$\:{F}_{R}=\sqrt{{{F}_{x}}^{2}+{{F}_{Y}}^{2}+{{F}_{Z}}^{2}}$$

$$\:{F}_{R}=\sqrt{{{15.35}^{2}+{4.97}^{2}+3.43}^{2}}$$

$$\:{F}_{R}=16.5\:kN$$

While the resultant force between X-axis and Z-axis (F_RXZ_) obtained are as below:

$$\:{F}_{RXZ}=\sqrt{{{F}_{x}}^{2}+{{F}_{Z}}^{2}}$$

$$\:{F}_{RXZ}=\sqrt{{{15.35}^{2}+3.43}^{2}}$$

$$\:{F}_{RXZ}=15.73\:kN$$

Afterwards, the angle of the resultant force for F_R_ ($\:\theta\:$) and F_RXZ_ (α) is define as:

$$\:\theta\:={tan}^{-1}\left(\frac{{F}_{Z}}{{F}_{X}}\right)$$

$$\:\theta\:={tan}^{-1}\left(\frac{3.43}{15.35}\right)$$

$$\:\theta\:=12.6^\circ\:$$

$$\:\alpha\:={cos}^{-1}\left(\frac{{F}_{RXZ}}{{F}_{R}}\right)$$

$$\:\alpha\:={cos}^{-1}\left(\frac{15.73}{16.5}\right)$$

$$\:\alpha\:=17.57^\circ\:$$

Due to constraints in testing equipment and safety considerations, the maximum applied load rate could not exceed a safety factor of 1.0. Hence, in this study, a safety factor of 1.0 was opted for the load applied in both normal and broken wire conditions. Nonetheless, it’s advised for subsequent investigations to adjust this safety margin to explore how increased loads affect the composite structure in the future. Consequently, in this research, the cross-arm was subjected to a 16.5 kN applied load at an angle of 12.6˚ from the horizontal plane and 17.57˚ from the vertical plane under the broken wire condition. The summarized working load and angle requirements for both normal and broken wire conditions, adhering to TNB Sdn. Bhd. specifications, are presented in Table 4^76^.

**Table 4:** Working load and angle specification.

Wire condition	Working load (kN)	Angle (˚)	
		From horizontal	From vertical
Normal	7.98	54.2	-
Broken	16.5	12.6	17.57

#### Modelling and finite element analysis

Finite Element Analysis (FEA) was conducted to simulate the deflection behavior of the pGFRP cross-arm under various loading conditions. Initially, the components of the cross-arm, including fittings, main cross-arm, tie cross-arm, screws, and nuts, were modeled using CATIA V5R18 software. Subsequently, the CATIA model was transferred to ANSYS FEA software for analysis, where the software applied equations governing the behavior of these elements and generated a comprehensive understanding of the system’s behavior^77^. To ensure the accuracy of the FEA results, it was assumed that the results converged to a solution and were independent of the mesh size. Mesh convergence analysis was carried out by adjusting the number of elements along the length of the cross-arm model, and the point at which the system’s response converged to the number of elements was considered the verified numerical result and was compared with experimental results.

For the FEA analysis of the load-deflection behavior, material properties listed in Table 3 were employed, maintaining identical geometry to the actual cross-arm, with dimensions of 102 × 102 mm² and 8.5 mm thickness for the square section. A medium mesh type and tetrahedron meshing method were selected to ensure accurate analysis results. Subsequently, a mesh convergence study was conducted with an element size of 30 mm, resulting in a total of 19,775 elements for the entire assembly, including fittings, main members, and tie members. During the simulation, one end of the pGFRP cross-arm beam was fixed while the load was incrementally applied at the other end, increasing in steps of 1000 N. This allowed the assembled cross-arm beam to deflect akin to a cantilever beam. The deflection results obtained from the FEA analysis were recorded and compared with experimental results for validation purposes.

### Experimental setup for flexural-creep test

**Fig. 7:**
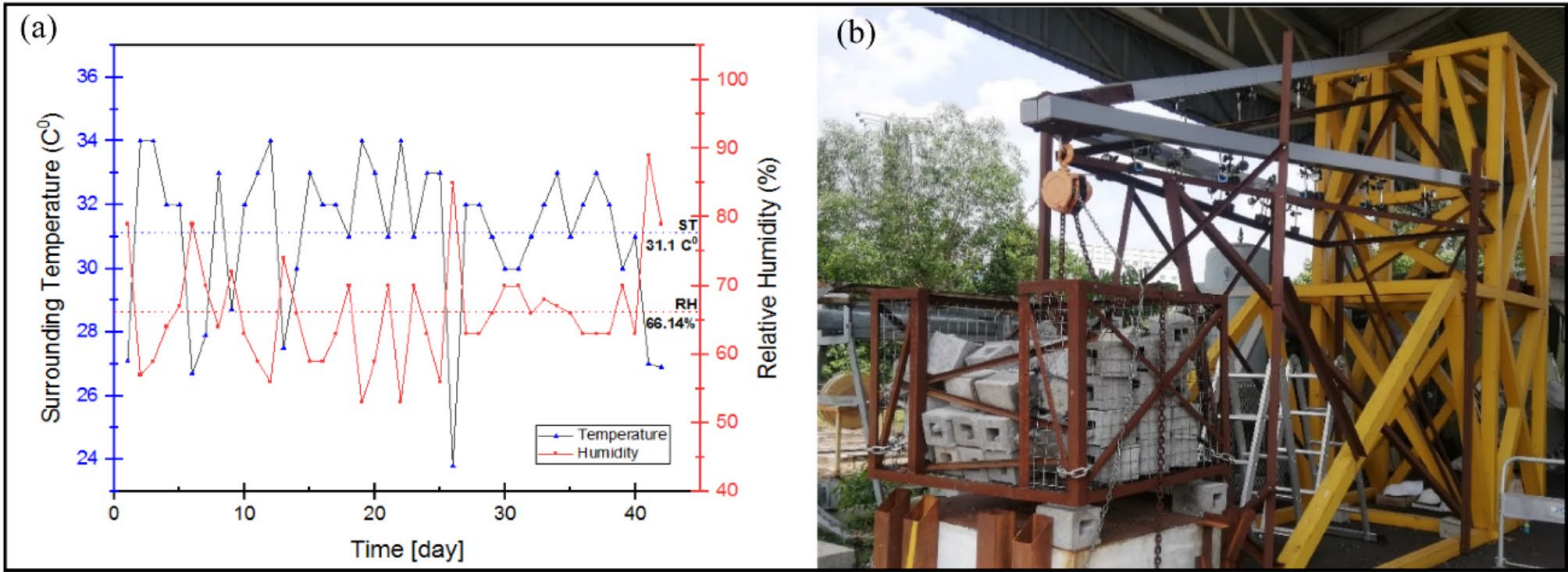
(**a**) Records of the ambient temperature and relative humidity throughout the creep test (**b**) Configuration of the cross-arm layout during creep test.

The complete actual-scale assembly cross-arm creep test was conducted with a load suspended at the free end of the cross-arm as depicted in Fig. 7. This test lasted for 1,000 h in an open area at Faculty of Engineering, Universiti Putra Malaysia, adhering to ASTM D2990 standards. Dial gauges placed at specific points (labeled as Y1 to Y5) across each cross-arm element measured deflection, with point Y1, nearest to the free end, set at a gauge gap of 0.61 m. Deformation values were recorded at various time intervals ranging from 0 to 1,000 h. To measure the load applied, a 3-ton crane scale was used at the unfixed end of the cross-arm, adjusted to 6467 N to simulate standard working conditions as per TNB standards^76^. The test exposed the cross-arms to genuine environment at tropical weather condition. Results included creep strain values, modulus comparisons, and quantitative analyses of creep for both existing and enhanced cross-arm designs. Testing was conducted outdoors with ambient temperature recorded at 31.1 °C and relative humidity at 66.14%, as shown in Fig. 7.

#### Lattice cross-arm properties

**Fig. 8:**
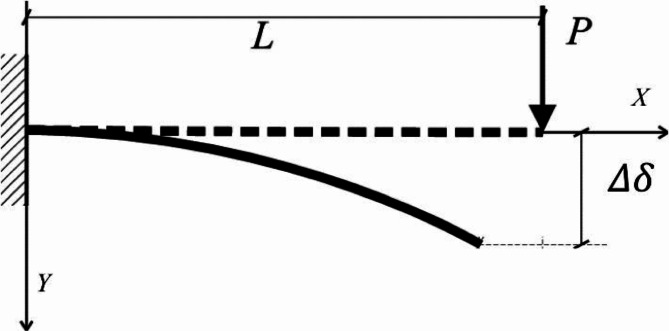
Schematic diagram for a cross-arm structure which obey cantilever beam concept.

The assumption was made that the connection between force and deflection of the cross-arm beams remained linear, provided the minor deflection and the beam material did not yield^78^. Because the material of the cross-arm under examination is pGFRP, with uniform density throughout its length, the proven and established Hooke’s Law from physics was applied, which asserts that force is directly proportional to extension^50^. Hence, the stress-strain correlation for the cross-arm is expressed by Eq. (1), illustrated in Fig. 8.

1 $$\:P=k\delta\:$$

Here, P represents the force exerted on the beam (in Newtons), k denotes the elastic coefficient (in Newtons per meter), and δ signifies the deflection (in meters). The elastic coefficient k correlates with the elastic modulus Ee, moment of inertia I, and beam length L. Employing the square-shape profile for the moment of inertia formula, where $\:{b}_{e}\:$and $\:{h}_{e}\:$were used as a width and length, Eq. (1) can be reformulated as follows:

2 $$\:{E}_{e}=\frac{4P{L}^{3}}{\delta\:{b}_{e}{{h}_{e}}^{3}}$$

The stresses $\:\left(\sigma\:\right)$ and strain $\:\left(\epsilon\:\right)$ through the beam align with the Hooke’s Law^79,80^ can be demonstrate as Eqs. (3) and (4).

3 $$\:\sigma\:=\frac{P(L-x)\frac{{h}_{e}}{2}}{I}=\frac{6P(L-x)}{{b}_{e}{{h}_{e}}^{2}}$$

4 $$\:\epsilon\:=\frac{\sigma\:}{{E}_{e}}$$

Basically, the cross-arm member encounters its peak stress at the anchored point (x = 0) and its lowest stress at the loading end (x = L).

#### Creep model for lattice cross-arm

The investigation into the cross-arm creep properties of pGFRP was expanded by utilizing Findley’s power law model, which has been validated in previous studies^12,81,82^. This model elucidates the transient creep behavior in relation to stress and material properties. While widely relevant, its computational process can be straightforward yet constraining. Moreover, the cross-arm was considered to be an anisotropic material. Consequently, the Findley model^81^ was employed to replicate the creep behavior and was expressed by Eq. (5).

5 $$\:\epsilon\:\left(t\right)={At}^{n}+{\epsilon\:}_{0}\:$$

Here, A and n represent the time exponents, while transient creep strain is denoted by ε, and $\:{\epsilon\:}_{0}$ stands for the immediate strain upon the application of load.

## Results and discussions

### Load-deflection under normal wire condition

**Fig. 9:**
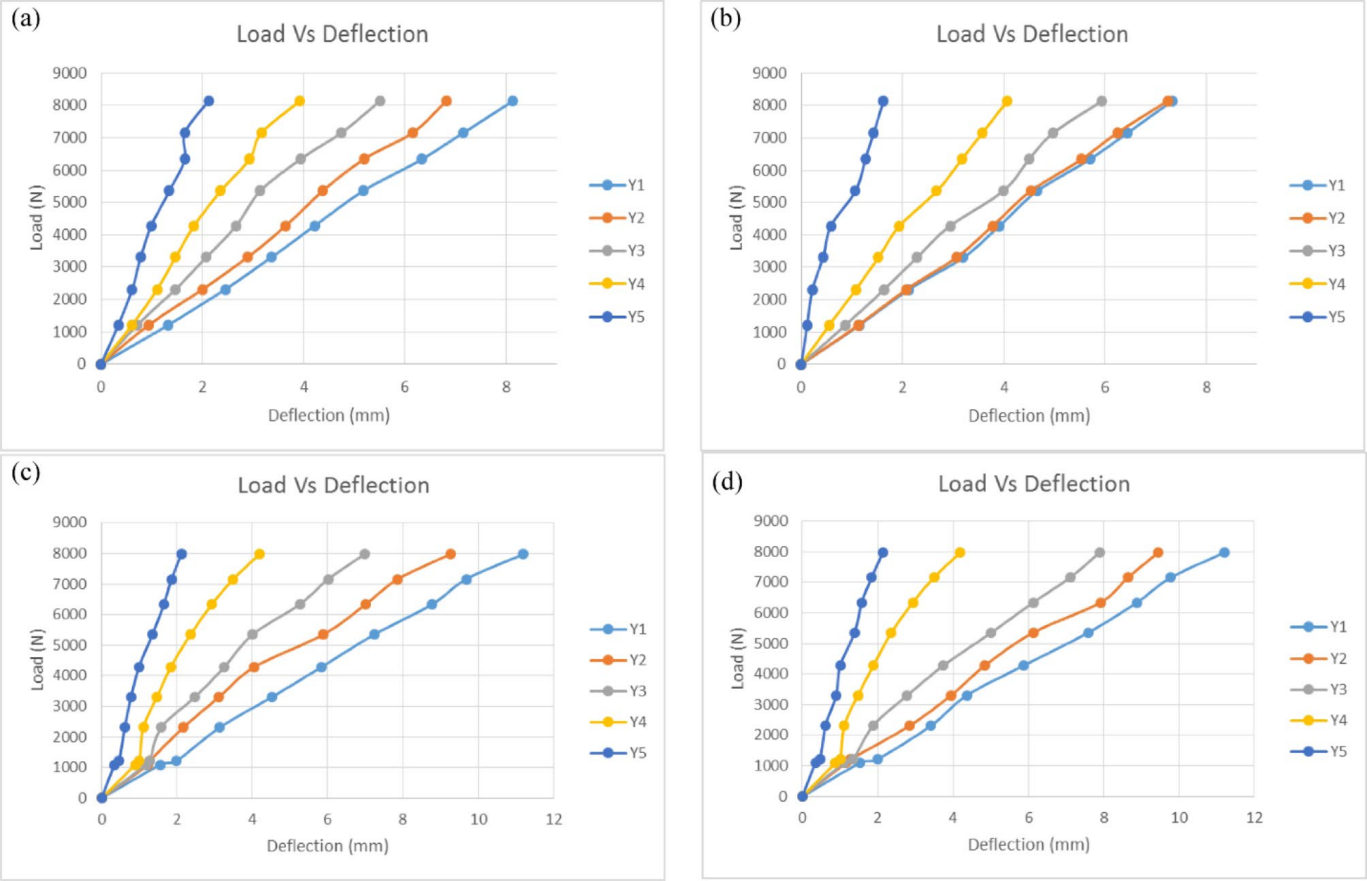
Load-deflection of pGFRP and wood cross-arm members in Y-axis for normal wire condition; (**a**) Main 1 pGFRP cross-arm members, (**b**) Main 2 pGFRP cross-arm members, (**c**) Main 1 wood cross-arm members, (**d**) Main 2 wood cross-arm members.

Figure 9 illustrates the load-deflection characteristics of Main 1 and Main 2 pGFRP and Balau wood cross-arm members under typical wire conditions along the Y-axis. The plot demonstrates a linear relationship between the applied load and deflection. Both materials behaves linearly under small deflections and as long as it doesn’t yield, following Hooke’s Law^83^. Maximum deflection consistently occurs at the free end, while minimum deflection occurs at the fixed point. The cross-arm’s deflection analysis resembles that of a cantilever beam and involves a three-dimensional problem. Poisson’s ratio influence can be disregarded due to specific geometric considerations^84^.

Figure 9a and b illustrate that the maximum deflection for the Main 1 pGFRP cross-arm member was below 9 mm, while for the Main 2 cross-arm member, it remained under 8 mm. In contrast, the Balau wood cross-arm members showed maximum deflections exceeding 11 mm for both Main 1 and Main 2, consistent with findings from previous research^76^ as depicted in Fig. 9c and d. The previous study by Yujun Qi also proved that under a transverse load, the deflection behaviour of wood was higher compared to GFRP profile due to high bearing capacity and flexural rigidity of the material^85^. Additionally, the deflection for both pGFRP and Balau wood cross-arm members in Main 1 increased uniformly along their lengths, whereas the Main 2 cross-arm members showed uneven deflection due to the applied angle relative to the horizontal plane. At point Y2, the deflection for the pGFRP cross-arm was slightly higher than at point Y1, likely because the applied angle intensified the force on Main 2 compared to Main 1. This angle also induced twisting in the Main 2 cross-arm, particularly at point Y2, leading to a steeper slope in the deflection graph for Main 2 compared to Main 1. Despite these differences, both pGFRP and Balau wood cross-arm members demonstrated a linear relationship between applied load and deflection.

Several researchers have used the same methodology used in this work to study the deflection behaviour of cantilever beams in various loading conditions using numerical and mathematical modelling^86–88^. C.M.Wang et al.^89^ has proven that the magnitude of deflection behaviour of a beam is dependent on the load position on the beam. The deflection increase as the distance between the applied load and the fixpoint increase, reaching its maximum value when the load is applied at the free end of the beam. As shown in Fig. 9, the higher deflection always occur at point Y1 as it was the nearest point to the free end of the cross-arm. The classical beam theory was used by H.J.Barten et al. and T.Balendez et al.^83,90^ in computing a mathematical model for the deflection of a cantilever beam of varied stiffness. The results had proven the linear relationship between the load and the deflection. Besides, the study on non-linear applied load angle of the cantilever beam is required to understand their behaviour as convinced by several researchers^91,92^. However, this research only focuses on the deflection behaviour and not on the applied load angle since the specification was given by the supplier.

**Fig. 10:**
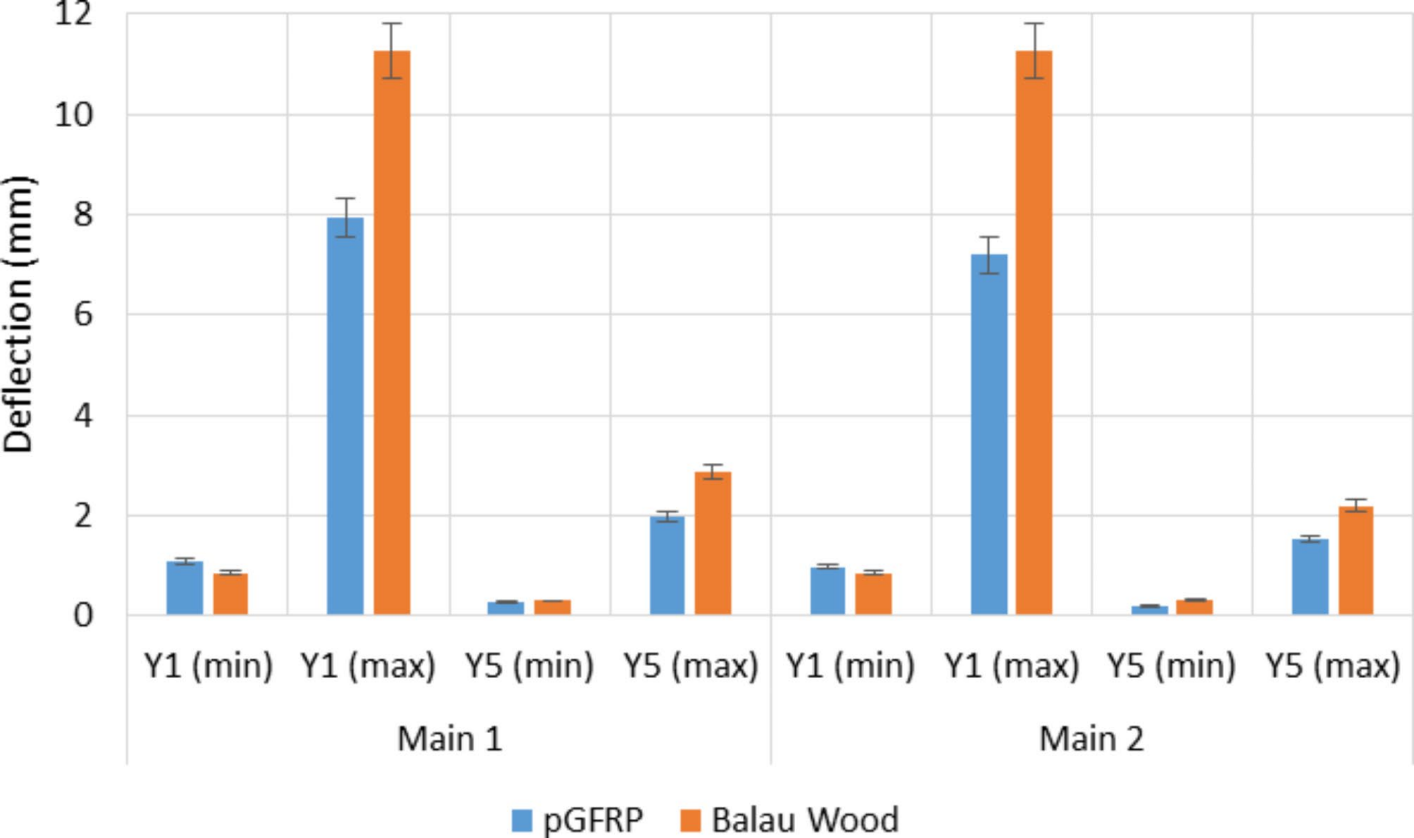
Difference of deflection value of pGFRP and Balau wood cross-arm in normal wire condition.

The deflection values of pGFRP cross-arm obtained at points Y1 and Y5 for the minimum of 1000 N and the maximum load of 7980 N were compared to those of Balau wood cross-arm, which was evaluated by Hussein et al.^76^. The difference in deflection values of both cross-arm beams at Y1 and Y5 is shown in Fig. 10.

It has been observed that the deflection values were slightly larger for both Main 1 and Main 2 pGFRP cross-arm members when the minimum load was applied at point Y1 as compared to those of the Balau wood cross-arm beam. However, the difference in deflection values was small at point Y5 for both cross-arm types, which was approximately 0.12 mm. On the other hand, in the working load condition, the deflection value of the Balau wood cross-arm beam was significantly higher compared to that of the pGFRP cross-arm members, where the deflection value difference between these two types of cross-arm was almost 3.7 mm. The main reason for this observation was the higher elastic modulus and flexibility of Balau wood compared to the pGFRP material^93^. Investigating how cross-arm beams deflect under working loads is essential for assessing the durability of high transmission towers.

Figure 10 illustrates the superior ability of the pGFRP cross-arm to withstand bending stress compared to the Balau wood cross-arm. In the construction of the pultruded composite cross-arm, a pultrusion process involving ten layers of ± 45° and 0° glass fibers reinforced with UPE matrix was utilized, as depicted in Fig. 2. The alternating stacking sequence between + 45° and − 45° at the outermost and innermost layers effectively resists shear strain caused by torsional loading from the cable, as indicated by Syamsir et al.^66,94^. The difference in the lamination sequence, ranging from 0° to 45°, led to an increased presence of continuous roving in the profile manufacturing process, thus influencing these results^95,96^.

Incorporating 0° outer layers on both sides of the pultruded composite aligns the principal loading direction with the ply orientation, as observed by Hashim et al.^97^. This configuration enables orthogonal exposure of the pGFRP cross-arm, thus improving its bending strength^98^. Alternating between 0° and ± 45° ply orientations results in variable stiffness throughout the laminate thickness, delaying glass fiber breakage and safeguarding the composite^99–101^. The UPE matrix’s macromolecular chain exhibits greater elasticity, further delaying glass fiber rupture, thus enhancing the composite laminate’s toughness and rigidity. Additionally, the use of UPE as a matrix in the composite laminate is compatible with E-glass fiber.

Although the deformation values of Balau wood and pGFRP cross-arms were differentt, both materials exhibited almost the same quality in resisting elastic deformation. The difference in the deflection values between Main 1 and Main 2 cross-arms members was probably due to the alignment angle after the load was applied. It should be noted that the load was applied manually and was dependent on inconsistent manpower energy. Thus, it is suggested to use the automated system during load application to improve the angle of cross-arm from the horizontal plane and to reduce the difference in deflection value between Main 1 and Main 2 cross-arms members. Figure 10 also shows that wood possesses lower mechanical properties compared to pGFRP due to the composition of natural fibre that consist of cellulose, hemicellulose, lignin and pectin^102^. This composition results in poor fiber interaction, leading to internal defects and cracks, consequently causing early crack propagation.

**Fig. 11:**
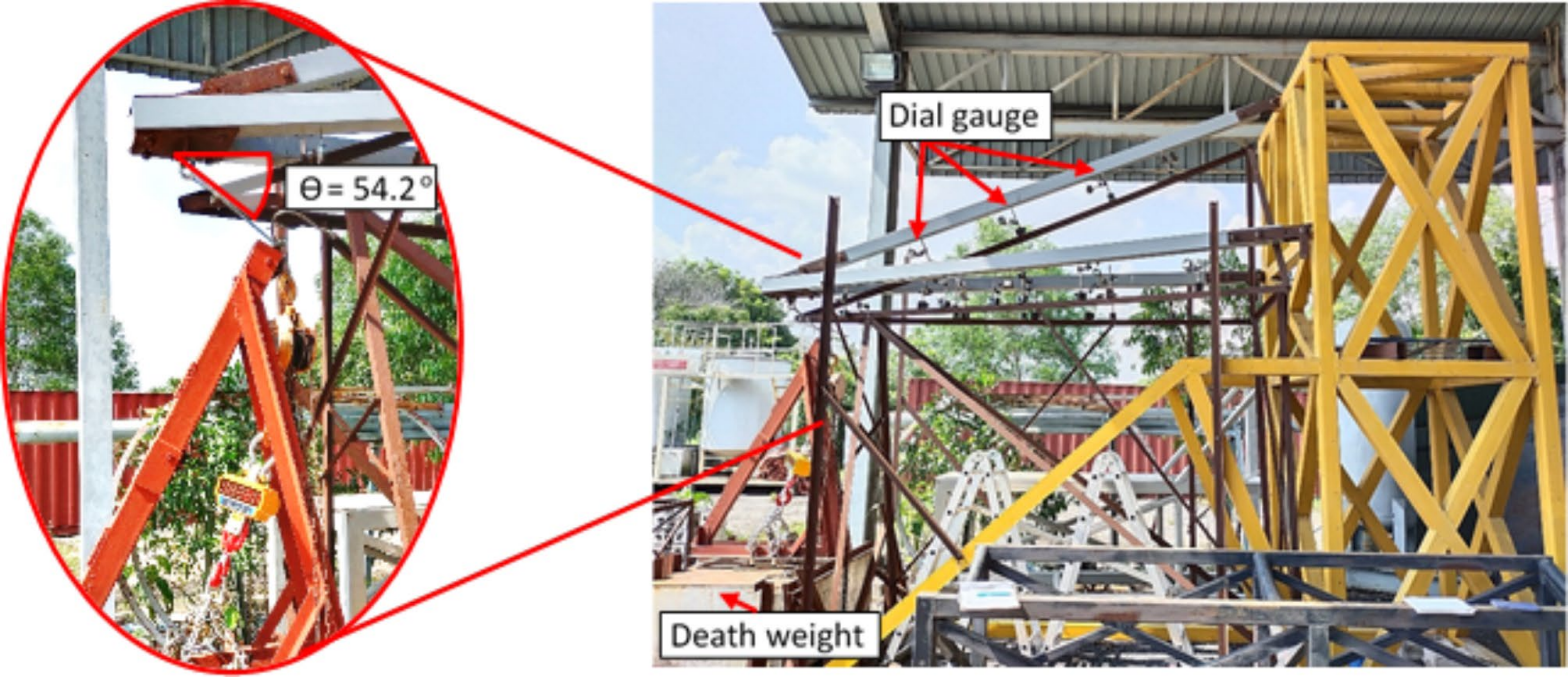
Actual pGFRP cross-arm position during normal wire condition study.

Moreover, experimental research demonstrated that pGFRP exhibits superior bending characteristics when compared to Balau wood. This improvement may be attributed to the pultrusion process, which ensures thorough wetting of the glass fiber by resin, thus minimizing void formation within the composite laminate^103,104^. The actual position of the cross-arm during load application was shown in Fig. 11. Hence, it can be concluded that the use of pGFRP instead of Balau wood in the fabrication of cross-arm structures for high transmission towers is advantageous in terms of bending strength and modulus.

### Load-deflection under broken wire condition

The load-deflection behaviour of cross-arm in broken wire condition was studied using the same method used in normal wire condition but with different angles from the horizontal and vertical planes. The results obtained for Main 1 and Main 2 cross-arm members in this condition were compared with the previous results obtained by Hussein et al. on the Balau Wood cross-arm structure^76^, which had similar profiles and dimensions. By using a 1.0 safety factor, the actual working load of 16,500 N was applied on the cross-arm at an angle of 12.6˚ from the horizontal plane and 17.57˚ from the vertical plane.

**Fig. 12:**
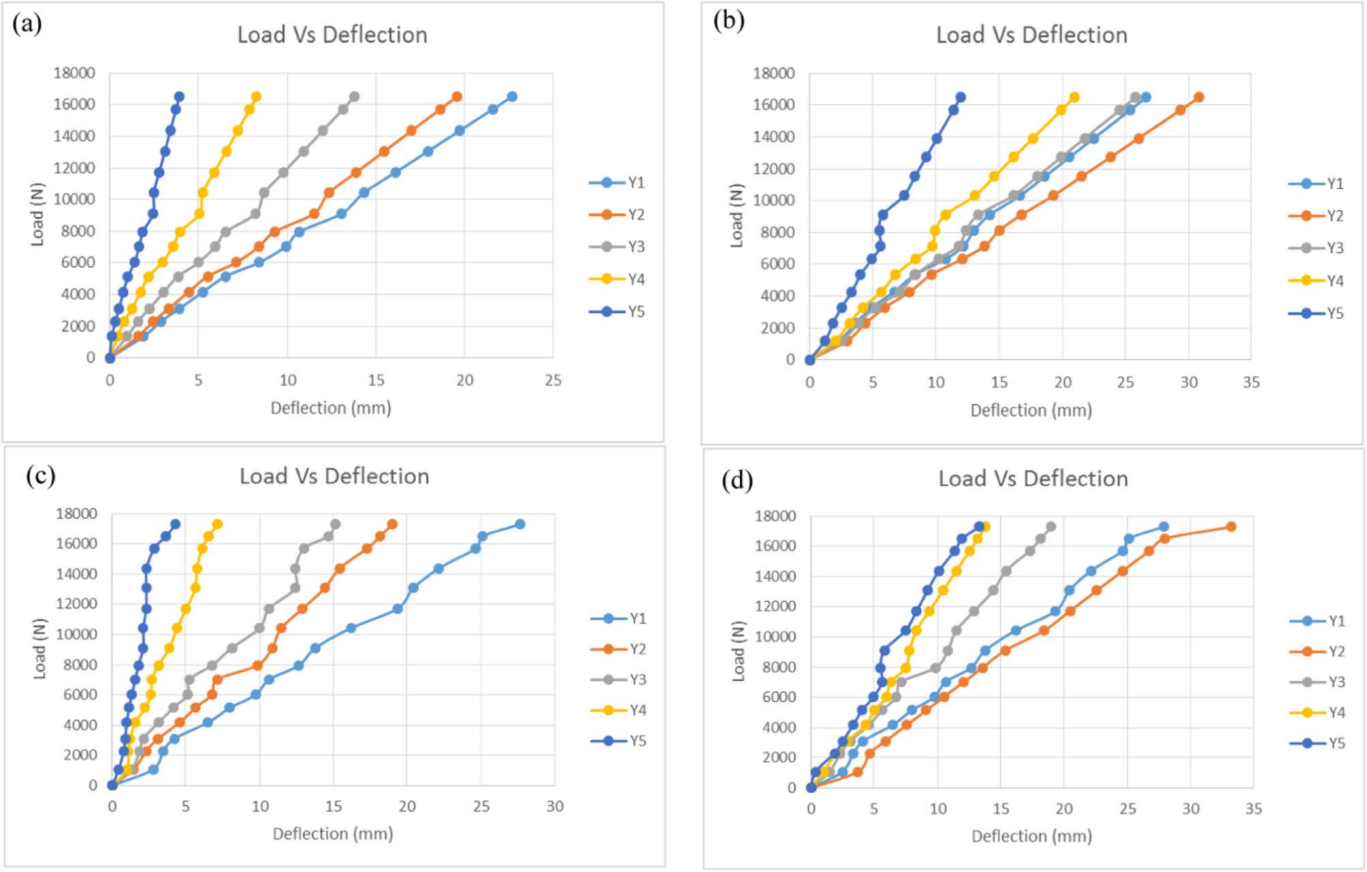
Load-deflection of pGFRP and Balau wood cross-arm members in Y-axis for broken wire condition; (**a**) Main 1 pGFRP cross-arm members, (**b**) Main 2 pGFRP cross-arm members, (**c**) Main 1 wood cross-arm members, (**d**) Main 2 wood cross-arm members.

Figure 12 shows the load-deflection behaviour of the Main 1 and Main 2 pGFRP and Balau wood cross-arm members subjected to actual working load in broken wire conditions. The deflection value and the applied load for the both pGFRP and Balau wood cross-arm were in a linear relationship similar to the normal wire condition. All considerations and properties discussed in Section “Load-deflection under normal wire condition” were used in obtaining the results in this condition. The broken wire condition mimics the actual position of the cross-arm with broken wire. Thus, the working load applied was 52% higher than the load in normal wire condition, which was 16,500 N. In this study, the load was increased gradually from 0 N to 16,500 N by steps of 1000 N. From this research, it was found thath, the pGFRP composites materials, can exhibit complex deformation patterns under different loading conditions, which must be thoroughly investigated to ensure their reliable performance including broken wire condition of the cross-arm. The cross-arm deflection during broken wire condition will be higher compare to normal condition due to the position of the broken wire and weather conditions like wind and surrounding temperature^105^. Other findings highlighted the necessity of accounting for complex seismic factors such as multicomponent excitations, wave passage, and coherency loss effects in the design and analysis of transmission line structures to ensure resilience against dynamics load such as in broken wire conditions^106^. In contrast, wood, a natural and versatile material, has been used in construction for centuries, although its deflection behavior is well-documented in the literature, however, there are limited study can be found on the deflection behaviour in broken wire condition.

The study highlights notable differences in deflection behavior between the Main 1 and Main 2 members of pGFRP and Balau wood cross-arms under applied loads. For the pGFRP cross-arm, the maximum deflection recorded was approximately 22.68 mm for Main 1 and 26.66 mm for Main 2. Main 1 displayed uniform deflection along its length, whereas Main 2 exhibited uneven deflection, likely due to the influence of the tested angle in the cross-arm structure. This unevenness was particularly evident at point Y2, where deflection values consistently exceeded those at point Y1, despite adherence to the supplier’s specifications for angles in the broken wire condition. This outcome suggests that the applied angle caused an imbalance in force distribution, increasing the load on Main 2, leading to twisting at point Y2, and ultimately reducing the deflection slope compared to Main (1) Similarly, Balau wood cross-arms demonstrated higher deflection values than pGFRP cross-arms, with 27.654 mm for Main 1 and 27.891 mm for Main (2) While Main 1 exhibited uniform deflection along its length, Main 2 mirrored the pGFRP pattern with greater deflection at point Y2. These results align with previous studies and underscore the impact of material properties and structural geometry on deflection behaviour for the cross-arm materials (M. R. M. Asyraf et al., 2021b; Sharaf et al., 2020). Despite these variations, both Main 1 and Main 2 for pGFRP and Balau wood maintained a linear relationship between applied load and deflection, reaffirming the predictability of their structural performance under increasing loads.

Typically, broken wire scenarios replicate the sudden breakage of wires within a transmission line, inducing immediate imbalances in forces within the system. These resultant loads can be substantial, potentially causing failure in either the cross-arm or the entire support structures. Various researchers have conducted static analyses of broken wire conditions to assess residual wire tensions and the loads exerted on support structures^107–110^. John et al.^111^ presented the analysis results of broken wire conditions and provided static and dynamic data on the longitudinal loading and structure response using prototype line systems. The corresponding static displacement and member forces acting on the cross-arm were measured and analyzed using the static analysis computer program. By using the equilibrium equations, John et al. derived an equation for insulator cable displacement by substituting the stiffness formulation into the force equilibrium relationships at each wire attachment^112^. The various angles during load applications due to broken wire played a role in determining the displacement of the cross-arm structures.

**Fig. 13:**
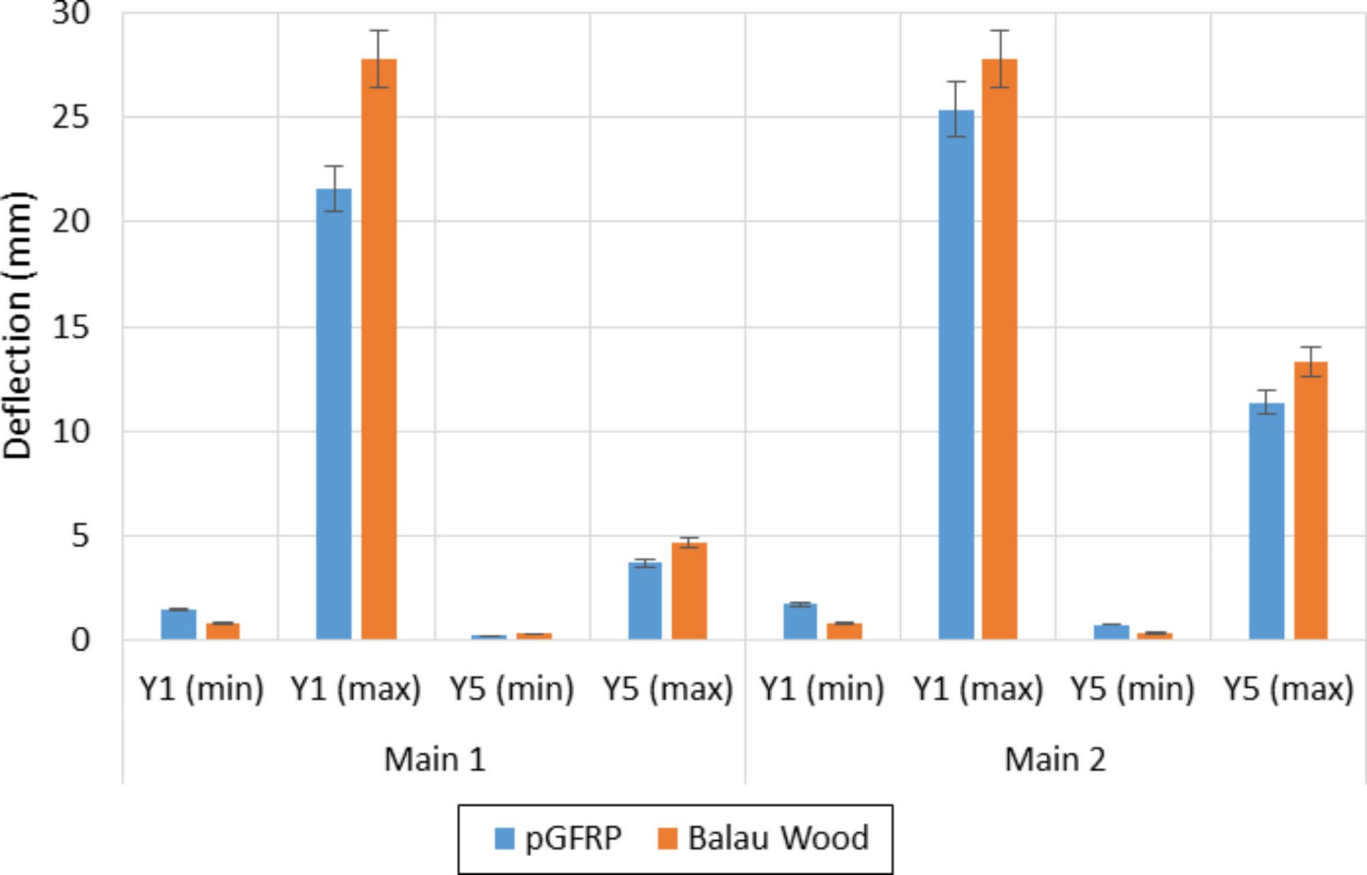
Difference of deflection value of pGFRP and Balau wood cross-arm in broken wire condition.

The maximum and minimum deflection of pGFRP cross-arm obtained at point Y1 and Y5 were compared with the results obtained by Hussein et al.^76^. The difference in deflection values for both cross-arm beam types in broken wire conditions at Y1 and Y5 is shown in Fig. 13.

It can be noted that the deflection values were slightly higher for both Main 1 and 2 pGFRP cross-arm members during the application of the first load of 1000 N at point Y1 as compared to the Balau wood cross-arm beam. However, the deflection value difference was considered small at point Y5 for both types of cross-arm members, which was around 0.43 mm. The pGFRP cross-arm responded immediately once the first load was applied and the deflection became stable after the working load was applied, where the deflection value was slightly higher at the beginning of the load application.

In the working load condition, the deflection value increased drastically for the Balau wood cross-arm compared to the pGFRP cross-arm, specifically for the Main 1 cross-arm member. For the Main 2 cross-arm member, the deflection values at points Y1 and Y5 became higher than those of the Main 1 cross-arm member due to the applied angle from the horizontal and vertical planes, which caused the cross-arm to be twisted in the direction of the angle. The structural impact of wire failure depends on its position relative to shear and flexural load zones. Near-support wire breaks maintain partial residual prestressing, while midspan breaks nearly eliminate prestressing effects. Structures with minimal transverse reinforcement are particularly vulnerable to brittle shear failures when wires break in shear spans^113^. As mentioned in Section “Load-deflection under normal wire condition”, the load was applied to the beam manually and thus, inconsistency may arise. However, this study focuses only on obtaining basic information on the deflection behaviour of the existing pGFRP cross-arm and compared them to that of Balau wood cross-arm. Thus, the twisting moment and torsional buckling were not discussed.

The difference between these two types of cross-arms was caused by Balau wood’s higher elastic modulus and flexibility when compared to pGFRP material. The pGFRP cross-arm, on the other hand, can sustain more bending stress than the Balau wood cross-arm, and the pultruded technique provided value in terms of reinforcing the composite structure. In addition, as the number of symmetrical broken wires increases, the horizontal cable tension decreases linearly in segments and the in-plane natural vibration frequency decreases linearly in segments. For shorter cables consideration, broken wire damage has a pronounced effect on static characteristics of the structure^114^. While the deformation values of the Balau wood and pGFRP cross-arms were varied, both materials demonstrated about the same resistance to elastic deformation. Under both normal and broken wire conditions, the linear relationship between load and deflection was the same. This understanding will serve as the foundation for future advancements in the cross-arm application for high transmission towers.

### Previous finding for the cross-arm deflection behavior

**Fig. 14:**
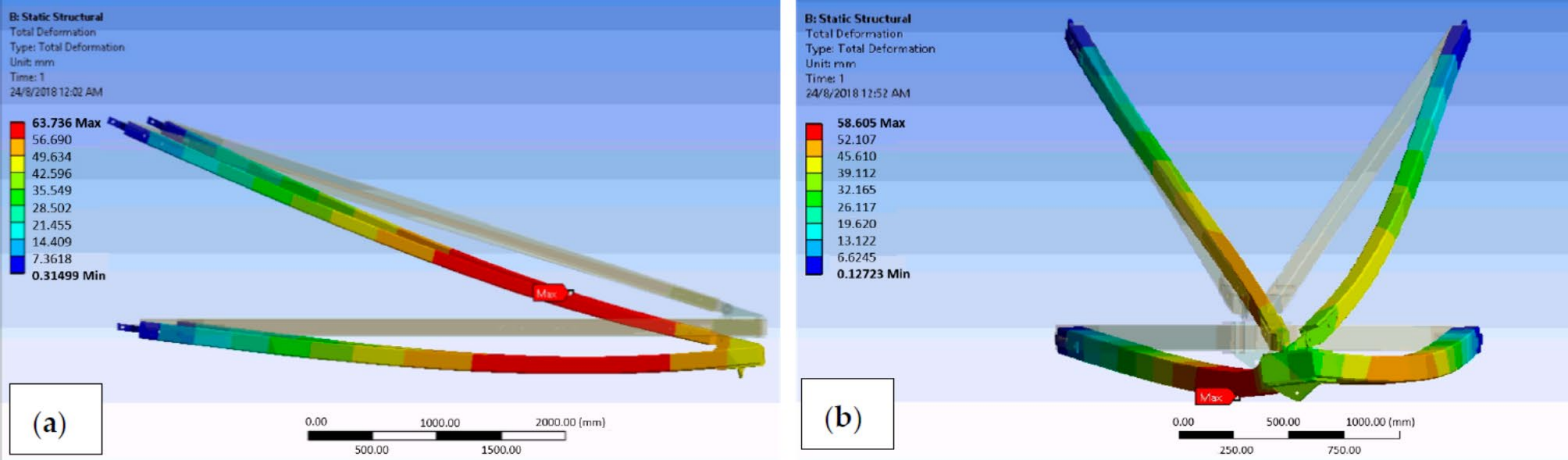
FEA for pGFRP Cross-arm deformation conditions of (**a**) normal and (**b**) broken wire^115^.

A numerical simulation was previously conducted to anticipate the stress, mechanical deformation, and failure safety factor of a composite cross-arm assembly consisting of four members within a transmission tower. The study, conducted by Al-Hayek et al.^115^, found that the outer ply of the main components of the composite cross-arm exhibited the highest critical value in a broken wire scenario, leading to lateral torsional buckling and deflection of the cross-arm, as shown in Fig. 14. The investigation’s results indicated that delamination could potentially cause failure in a damaged wire scenario, as depicted in Table 5.

**Table 5:** pGFRP cross-arm failure safety factor for each layer^115^.

Loading Condition	Fibre, s1	Matrix, s1	In-Plane shear, s12	Out-of-plane shear, s13	Delamination, s3
Normal	4	2.1	1.4	1.4	7.98
Broken	4	1.2	3.5	2	16.5

**Fig. 15:**
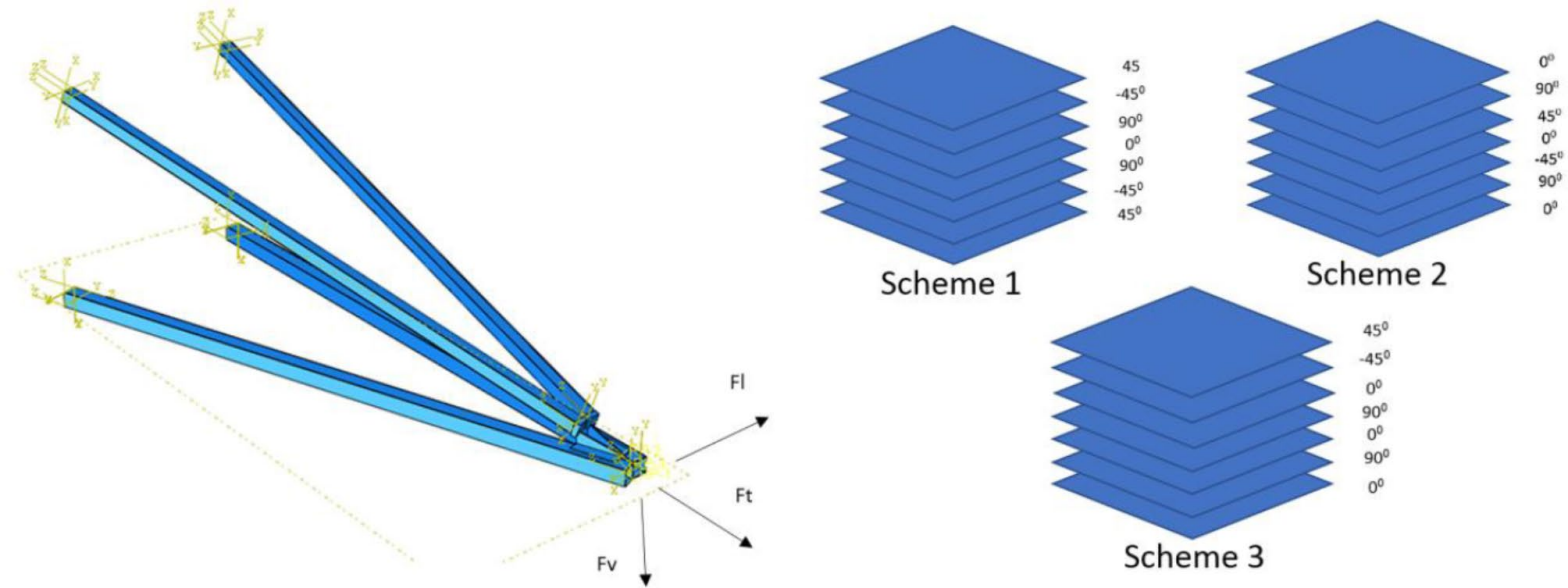
The composite cross-arm’s schematic for the sequence of fabric layers^116^.

Mohamed et al.^116^ undertook research to investigate the impact of composite layer arrangement on the behavior of the cross-arm structure under multiaxial quasi-static loading. However, as shown in Fig. 15, the distribution of layers with different fiber orientations significantly influenced the static displacement of the structure. Difference research conducted by Mohamed et al.^19^ examined the effect of laminate properties influence the failure under multi-axial pressure of composite cross-arm structures. This research assessed three different lamination patterns to examine the failure behavior and deflection characteristics of the composite cross-arm.

On the other hand, the arrangement of layers had minimal impact on the static displacement of the structure. Nevertheless, research indicates that failure in the cross-arm laminate results from both the stacking order and the proportion of layers with distinct fiber orientations. Table 6 illustrates the maximum deflection of composite cross-arms with different fiber stacking sequences.

**Table 6:** Different stacking sequence effect on the deflection behaviour of the composite structure.

Scheme	Composite laminate lay-up	Maximum deflection, mm
1	[45/-45/90/0/90/-45/45]	189
2	[0/90/45/0/-45/90/0]	234
3	[45/-45/0/90/0/90/0]	245

In this point of view, the cross-arm with greater Young’s modulus and ultimate bending loads experiences only one mode of failure: fiber buckling, which happens at a deflection of 0.082 m under compression. However, the specific designs and properties of the laminate were unable to prevent the cross-arm from failing when exposed to multiaxial loading. Thus, this research revealed on the experimental results of load-deflection behaviour between pGFRP and Balau wood cross-arms. If the cross-arm did not yield, both composite and wooden cross-arms showed a direct correlation between the applied load and the deflection.It could be seen in the previous sections that the pGFRP cross-arm had a quick response towards the applied load in the early stage as compared to the Balau wood cross-arm in normal wire conditions and it stabilized as the applied load reached the working load.

The pGFRP cross-arm demonstrated higher stress resistance due to the application of 0° orientation on the outermost layers, serving as effective load distributors that evenly distribute force throughout the polymer matrix. The alternating ± 45°/0° sequences in the middle acted as energy absorbers^117–119^. Studies by Amaro et al.^120^ and Reis et al.^121^ highlighted that varying the thickness of ply layers in stacking sequences significantly enhances the energy-absorbing properties of polymer composites, thus prolonging the time before glass fiber fracture under applied force. Additionally, the presence of UPE as a matrix in the composite laminate contributed to increased toughness and stiffness, while the compatibility of E-glass fiber with UPE resin further reinforced these properties^122,123^.

However, the Balau wood cross-arm exhibited critical deflection behaviour during the application of the working load due to its lower mechanical properties and weak interaction among the fibres. This proves that the pGFRP cross-arm has overcome its predecessor cross-arm due to its good material strength and fabrication process. The pGFRP cross-arm results obtained experimentally in this research then compared with the FEA analysis was discussed in the next section.

### FEA model accuracy and validation

#### Mesh convergence study

A mesh convergence study was performed to verify the accuracy of the FEA model, namely to ensure that the FEA model captures the system behaviour and gives the most accurate data. By using an iterative method, the number of elements in the mesh was increased from 7000 to 32,600 along the model length and were solved. The complexity of the model and the response were recorded. The deflection at point Y1 of the model subjected to the working load of 7980 N was chosen as the response while the number of elements in the mesh became the complexity of the model^124,125^.

**Fig. 16:**
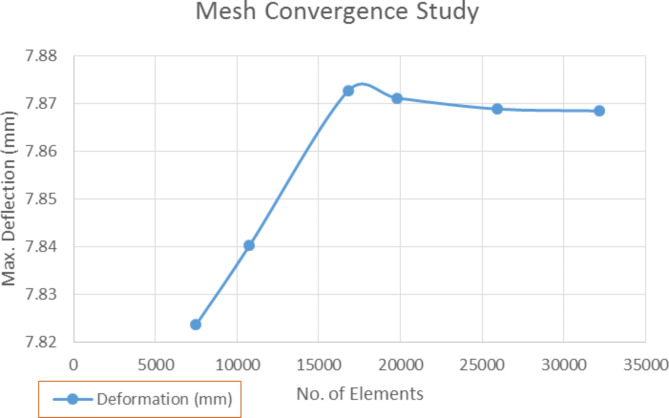
Mesh convergence study for FEA model.

**Fig. 17:**
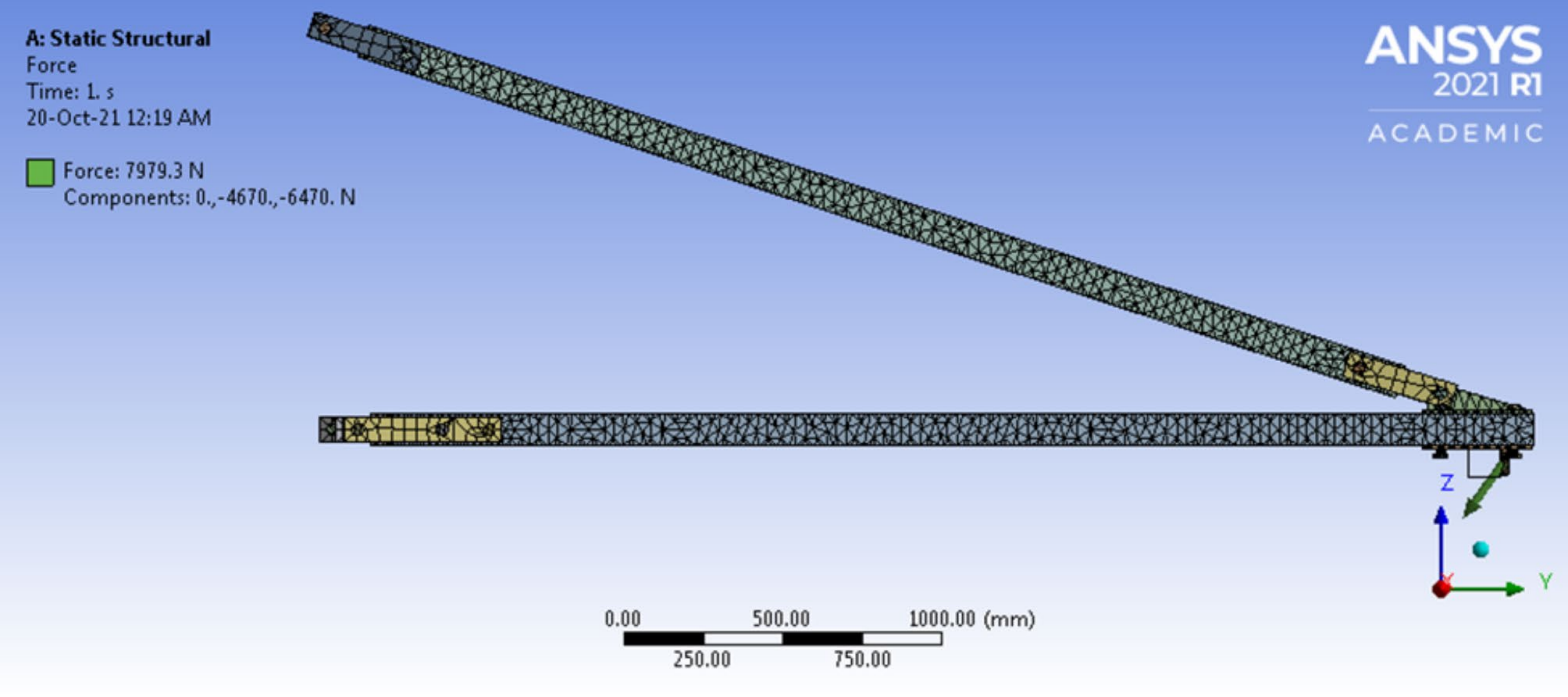
Meshes of pGFRP Cross-arm assembly.

Using the Tetrahedrons meshing method on the cross-arms and auto-meshing method on the fitting body (as shown in Fig. 17), the FEA model was meshed and their maximum deflection results were observed. Figure 16 presents the mesh convergence study of the FEA model. In the beginning, the maximum deflection increased linearly with the number of mesh elements. However, the graph started to converge and remained constant after reaching 25,000 elements. Furthermore, it was observed that no further variation in the deflection value occurred after 32,600 elements and this number of elements exhibited stable conditions as compared to other numbers of elements. Thus, 32,172 numbers of elements was chosen as a criterion for mesh convergence result that was to be used in FEA deformation analysis.

#### Validation of finite element analysis result

By implementing the cantilever beam concept, the experimental results of the pGFRP cross-arm were verified against the FEA results on deflection behaviour, where one end of the beam was fixed and the load was applied to the free end, which was gradually increased till achieved the working load condition. The force applied in the FEA model was the same as in the experiment. In this study, the normal wire condition was chosen to compare with the experimental results and for the deflection behaviour, the focus was on the maximum deflection occurring at point Y1.

**Fig. 18:**
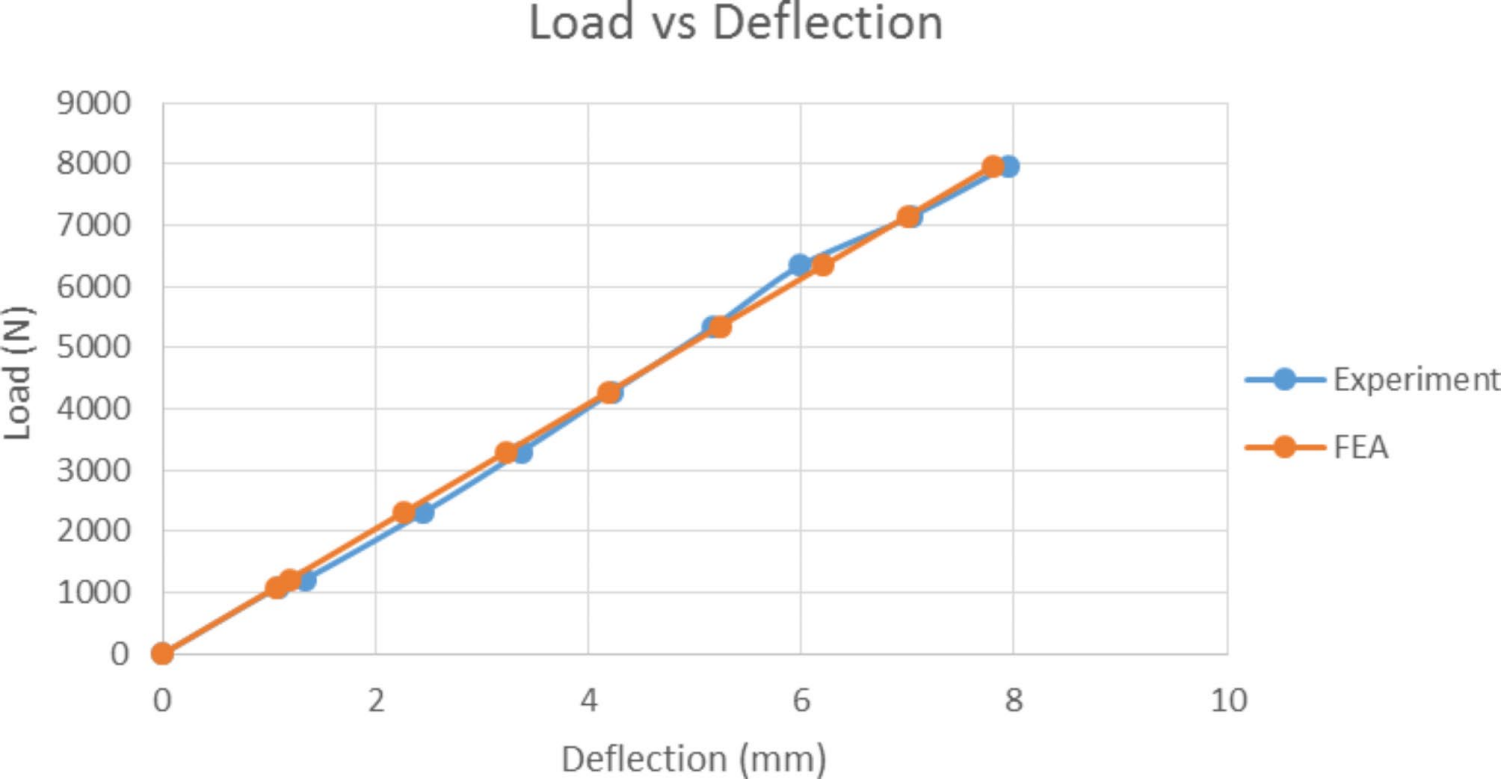
Result comparison between Experimental and Numerical analysis.

**Fig. 19:**
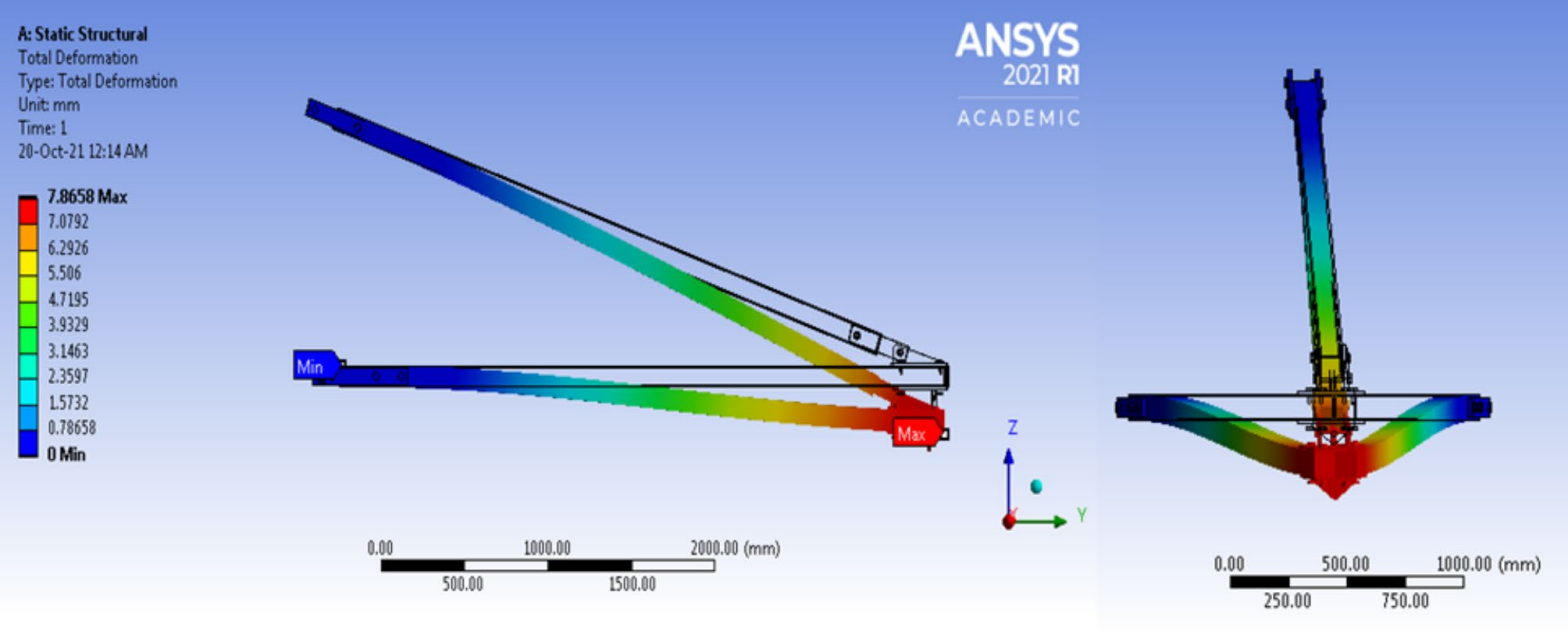
Deflection behaviour of Cross-arm’s FEA model.

Figure 18 shows that the deflection behaviours obtained from the experiment and FEA simulation had a linear relationship with the applied load. The FEA results were stiffer than the experimental results due to the assumption that the FEA model possessed perfect material bonding and the load was applied uniformly^126^. The maximum deflection under working load obtained from the experiment and FEA simulation was 7.939 and 7.866 mm respectively. The relative error between them was less than 1%, which was acceptable and complied with the cantilever beam concept. The difference in deflection values was probably resulted from the inconsistency that arose during the manual application of the load to the beam. Figure 19 shows the FEA results for total deflection of pGFRP cross-arm in normal conditions. From the images of deflected and not-deflected cross-arms shown in Fig. 19, it could be seen that the maximum deflection occurred at the free end while the minimum deflection occurred at the fixpoint.

### Lattice cross-arm flexural creep evaluation

**Fig. 20:**
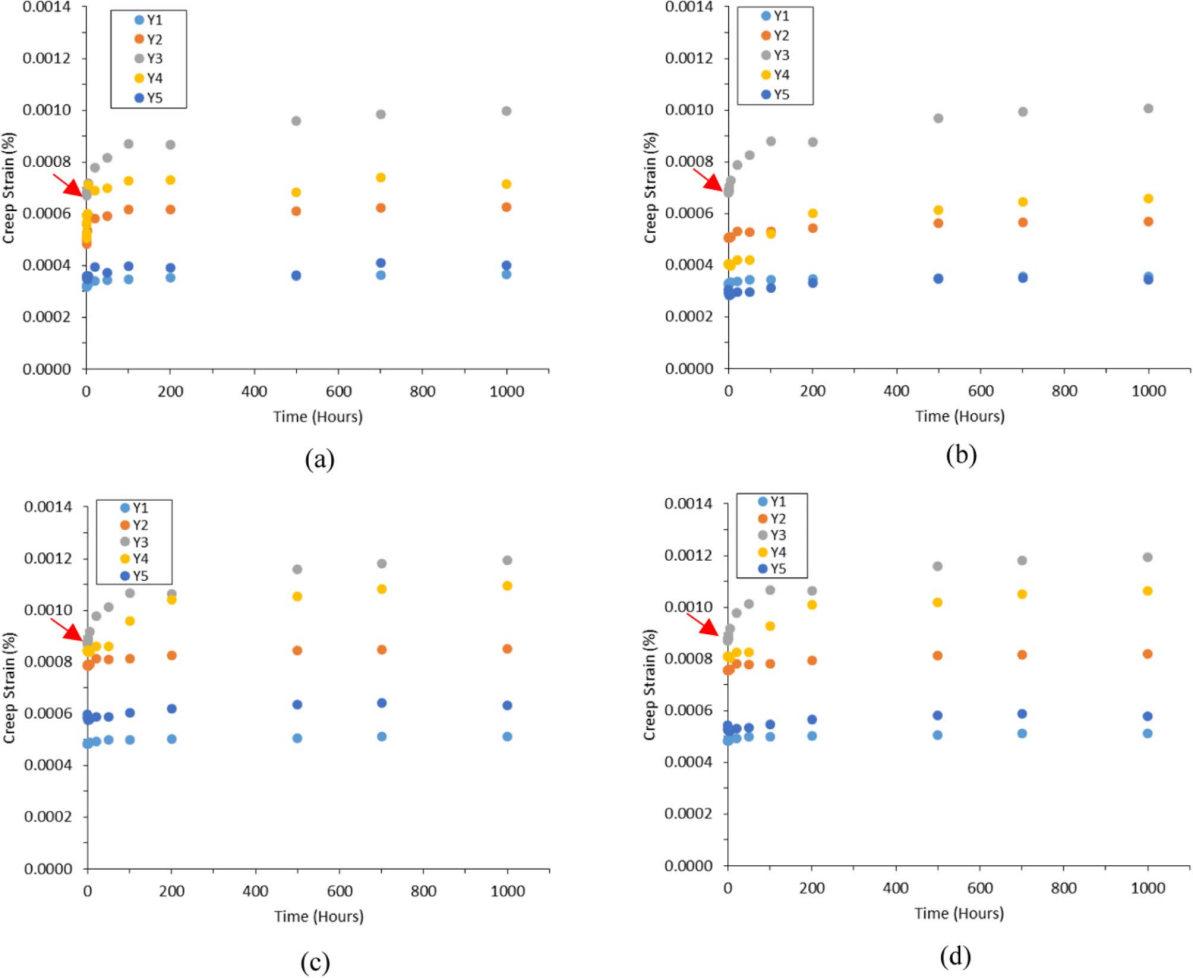
Creep strain for pGFRP and Balau wood cross-arm from experimental results (**a**) Main 1 pGFRP cross-arm member (**b**) Main 2 pGFRP cross-arm member. (**c**) Main 1 Balau wood cross-arm member (**b**) Main 2 Balau wood cross-arm member.

The attributes of the assembled pGFRP and Balau wood cross-arm were assessed using a two-point bending test, chosen for its resemblance to the behavior of a cantilever beam. This testing approach was adopted from previous studies conducted by Asyraf et al. and Hussein et al.^23,76^. The findings for the Main 1 and Main 2 composite cross-arm members in this study were compared with those of Balau wood cross-arms^127^. Given the similar profiles and dimensions of both cross-arms. Figure 20 illustrates the creep strain-time curves for composite cross-arms by applied actual operational loads at Points Y1 to Y5. Each dial gauge recorded a consistently the creep strain values, reflecting the tension and compression effects through the cross-arm member during load application^80^. As stress propagated along the beam from the loading point to the fixed end, elastic strain values varied along the length of the main member beams^128^. This investigation highlights that the highest creep strain occurred at Point Y3, a finding consistent with previous research^12^, which corresponds to the midpoint of the main members in the current and reinforced cross-arm design. Since the applied load was at the free end of the main cross-arm member beams, this outcome was attributed to beam buckling^129,130^.

Comparison of results between the Main 1 and Main 2 of both pGFRP and wood cross-arms revealed slightly differing creep strain levels due to asymmetrical jigging and fixing during installation. However, their creep strain curve patterns were largely similar, conforming to standard creep strain curves^131^, indicating the cross-arm’s ability to endure loading conditions. Despite potential misalignment issues inherent in the experimental setup mimicking real-world applications, it was evident that reinforcing the cross-arms structure using pGFRP could enhance their creep resistance compare to wood cross-arm^132^.

Figure 20 also demonstrates a transition in creep strain configuration for the cross-arm from elastic phase to viscoelastic stages. The red arrows indicate that the pGFRP cross-arm undergoes a more prolonged transition period compared to the wood cross-arm, suggesting a more stable viscoelastic stage and thereby reducing the risk of structural failure. The strain values at Point Y3, found to be larger in both pGFRP and wood cross-arms compared to other points along the cross-arm members (Points Y1, Y2, Y4, and Y5), highlight Point Y3 as a critical area. Therefore, future studies should focus on enhancing the cross-arm’s performance by reinforcing Point Y3 through the application of better reinforcement strategies, such as adding a bracing system, considering the use of a honeycomb sandwich structure, and exploring the utilization of winding basic concepts^45,133–136^.

Furthermore, the initial elastic modulus (Ee,_0_) and initial creep strain (ε_0_) were calculated using Eqs. 2 and 4 based on the observed creep deflection results under applied loads. The initial elastic modulus was directly correlated with the measured points along the cross-arm members, showing the maximum modulus occur at the fixed point (Point Y5) and the minimum occur at the free end of the cross-arm (Point Y1), as detailed in Table 7. The pGFRP cross-arm exhibited slightly lower initial creep strain at all points compared to the wood cross-arm but gradually became more stable over time due to the presence of glass fiber, which increased material strength and stabilized creep strain. In conclusion, enhancing the creep resistance of the cross-arm assembly against lateral forces resulting from dead weight, which may cause buckling, can be achieved by reinforcing the composite structure^74,137^.

**Table 7:** Result obtain from the experimental of pGFRP cross-arm.

Cross-arm member	Location	Stress, (MPa)	ε_0_, (10^− 4^)		$\:{\text{E}}_{\text{e},0}$ (GPa)	
			pGFRP	Balau wood	pGFRP	Balau wood
Main 1	Y1	97.29	3.1731	4.8295	306.62	201.45
	Y2	196.04	4.9722	7.8653	394.28	249.25
	Y3	294.79	6.7140	8.6914	439.07	339.17
	Y4	393.54	5.5815	8.4476	705.08	465.86
	Y5	492.29	3.5540	5.9756	1385.21	823.83
Main 2	Y1	97.292	3.2756	4.8295	297.2	201.45
	Y2	196.04	5.0479	7.5522	388.41	259.58
	Y3	294.79	6.8081	8.6914	433.00	339.17
	Y4	393.54	4.0478	8.1082	972.23	485.36
	Y5	492.29	3.0664	5.4252	1605.42	907.41

The stress at different locations on the cross-arm was evaluated using Eq. 3, taking into account the distance of each location from the fixed point of the cross-arm. Because both cross-arms share similar dimensions, the stress measured was uniform across all locations. As mentioned earlier, the dial gauges were positioned to maintain a consistent distance of 0.61 m between each location and the fixed point. Consequently, the effective lengths at Points Y1, Y2, Y3, Y4, and Y5 were 3.05 m, 2.44 m, 1.83 m, 1.22 m, and 0.61 m respectively. Table 7 illustrates that the stress experienced by the cross-arm member increased steadily from the free end to the fixed point, aligning with findings from previous studies^138^.

In Table 7, the initial creep strain for Balau wood cross-arm was higher compare to the pGFRP cross-arm. Additionally, Table 7 also indicated that the initial creep strain for Main1 and Main 2 cross-arm members for woods were almost exceeded 0.0009% specifically at Point Y3. However, the initial creep strain for pGFRP cross-arm were not even rich 0.0007% specifically at Point Y3. This indicated that both wood and pGFRP materials displayed similar abilities to withstand deformation elastically, on the other hand, the strength of wood was notably 34% lower compared to pGFRP, attributed to the inferior mechanical properties of hardwood in comparison to pGFRP^102,132^, particularly at the critical Point Y3. The inadequate mechanical characteristics of wood stem from its natural fiber composition, comprising cellulose, hemicellulose, lignin, and pectin. These components fostered limited interaction between fibers, leading to internal flaws and fissures. Consequently, this facilitated the premature initiation and expansion of cracks^139,140^. On the contrary, the pultruded GFRP composite utilized in the study displayed notably greater bending characteristics when compared to the GFRPC composite examined by Asyraf^12,132^. This disparity may be attributed to the pultrusion process, which facilitated thorough resin impregnation of the glass fibers, minimizing void formation in the composite laminate. Consequently, both the strength and modulus of these two cross arm materials hold considerable value for use in heavy construction transmission applications.

### Flexural creep model validation for cross-arm

The analysis of creep properties in cross-arm performance is integral to ensuring structural stability and reliability. Extensive testing of mechanical properties is necessary to qualify composite materials for various applications^141^. One established model used in this analysis is the Findley Power Law. The use of well-established models, such as the Findley Power Law Model, allows for a thorough assessment of parameters that affect creep behavior^23,142^ especially for assessing cross-arm performance. Table 8 illustrates the steady-state creep of the cross-arm as determined. The model facilitated the assessment of various parameters expressed by Eq. (5), which include transient creep A and the stress-independent material exponent n.

**Table 8:** pGFRP and Balau wood cross-arm transient creep (A) and stress-independent material exponent (n) according to Findley power law model.

Main Cross-arm Member	Location	A		N		Adj. R^2^	
		pGFRP	Balau Wood	pGFRP	Balau Wood	pGFRP	Balau Wood
Main 1	1	1.096 × 10^− 5^	3.241 × 10^− 4^	0.229	0.259	0.975	0.998
	2	5.415 × 10^− 5^	9.519 × 10^− 5^	0.149	0.333	0.943	0.963
	3	5.046 × 10^− 5^	6.197 × 10^− 5^	0.286	0.286	0.918	0.980
	4	7.488 × 10^− 5^	8.010 × 10^− 5^	0.158	0.417	0.901	0.920
	5	10.70 × 10^− 5^	1.198 × 10^− 5^	0.229	0.487	0.829	0.956
Main 2	1	5.253 × 10^− 6^	1.076 × 10^− 4^	0.259	0.259	0.918	0.988
	2	7.059 × 10^− 6^	1.059 × 10^− 4^	0.333	0.333	0.936	0.963
	3	5.046 × 10^− 5^	8.887 × 10^− 5^	0.286	0.286	0.910	0.980
	4	1.644 × 10^− 5^	5.463 × 10^− 5^	0.417	0.417	0.902	0.920
	5	2.289 × 10^− 6^	4.037 × 10^− 4^	0.487	0.487	0.865	0.945

The study compared how much the pGFRP and Balau wood cross-arms bend over time due to creep behavior as shown in Table 8. It was noted that the pGFRP cross-arm exhibited reduced transient creep in comparison to the Balau wood cross-arm across most points along the main members, attributable to its superior steady-state creep behavior. This transient creep strain represents the initial phase of inelastic flow, gradually diminishing until reaching a steady state^143^. It was found that the pGFRP cross-arm bent less overall because it’s mechanical properties was stronger than wood and has a superior creep resistance. The graph also indicates that the addition of glass fiber did not impact the secondary creep phase due to the reinforced resistance to creep in the pGFRP cross-arm. This initial bending which is transient creep was gradually slows down until it reaches a steady state for both type of cross-arm^42,144^.

The pGFRP composite cross-arm exhibits a lesser material stress-independent exponent, denoted as “n,” compared to the Balau wood cross-arm, with values of approximately 0.283 and 0.337 respectively (see Table 9). However, both the pGFRP and Balau wood cross-arms’ “n” values fall below the standard range of stress-independent material exponents^23,137,145^. Despite the improvements seen in load deflection and transient creep values when using pGFRP in cross-arm structures, as demonstrated in this study, further enhancements are necessary to improve the mechanical properties of the cross-arm. This could involve the implementation of measures such as introducing a bracing system^146^, utilizing sleeve structures^82^, employing winding methods^133^, and considering honeycomb sandwich structures^135,147^.

**Table 9:** Average material stress independent exponent, n for for pGFRP and Balau wood cross-arm.

Configuration	pGFRP Cross-Arm		Balau Wood Cross-Arm	
	Main 1	Main 2	Main 1	Main 2
Material stress independent exponent, n	0.210	0.356	0.281	0.394
Average	0.283		0.337	

Table 8 presents the adjusted regression (Adj. R^2^) values for Findley’s model applied to both pGFRP and Balau wood cross-arms. The Adj. R^2^ values for the pGFRP cross-arm ranged from 0.920 to 0.998, indicating a close proximity to 1, signifying the effective explanatory power of Findley’s model with the experimental data. In contrast, the Adj. R^2^ values for the Balau wood cross-arm fell between 0.829 and 0.975. These results indicate that the reinforced composite cross-arm followed the creep principle throughout the primary and secondary creep stages, and the data showed a reduction in exaggeration with increased structural integrity.

### Cross-arm structural behaviour under load application

The structural performance of cross-arms under applied loads is a crucial determinant of their reliability in high-stress applications, such as transmission towers. This study offers an in-depth analysis of the deflection and flexural creep behavior of pultruded glass fiber-reinforced polymer (pGFRP) and Balau wood cross-arms, focusing on their response to both normal and extreme loading conditions. Both materials exhibited a linear load-deflection relationship, adhering to the cantilever beam theory, with maximum deflection occurring at the free end and gradually diminishing towards the fixed end. However, pGFRP cross-arms consistently outperformed their Balau wood counterparts, demonstrating superior flexural rigidity, bending strength, and resistance to torsional stresses. These advantages are attributed to the optimized laminate structure of pGFRP, which employs alternating fiber orientations to evenly distribute loads and minimize internal stress concentrations. In contrast, the natural fiber composition of Balau wood was more susceptible to internal defects, crack propagation, and higher deformation under stress, making it less suitable for high-performance applications.

Finite element analysis (FEA) further validated the observed deflection behavior, highlighting the accuracy and computational efficiency of the model. A mesh convergence study established optimal simulation parameters, with results closely matching experimental data and yielding a relative error of less than 1%. This alignment underscores the reliability of the FEA model as a robust tool for predicting deflection behavior under various load conditions. Discrepancies between experimental and FEA results were minimal, primarily arising from perfect bonding assumptions in the simulations and variations in manual load application. The consistent performance of the pGFRP cross-arms across experimental and simulated evaluations reinforces their suitability for demanding structural applications.

The study also examined the flexural creep response of the cross-arms, providing critical insights into their long-term performance and structural optimization potential. Experimental data from two-point bending tests revealed that creep strain patterns conformed to standard viscoelastic behavior, with the highest strain observed at the midpoint of the main members (Point Y3), a critical stress concentration area. While both materials exhibited similar initial elastic deformation resistance, pGFRP cross-arms demonstrated superior creep resistance, transitioning more effectively into a steady-state creep phase and reducing the risk of long-term structural failure. These findings highlight the enhanced mechanical properties of pGFRP, including reduced internal flaws and improved resin impregnation, which contribute to its durability and reliability under sustained loads.

Further analysis using the Findley Power Law model validated the creep behavior of both materials, with pGFRP exhibiting lower transient creep and a smaller stress-independent material exponent (n) compared to Balau wood. The higher adjusted regression values (Adj. R²) for pGFRP confirmed the model’s accuracy in capturing its creep response. Despite these advantages, the study identifies opportunities for further enhancing the mechanical performance of pGFRP cross-arms, such as incorporating advanced design features like bracing systems, honeycomb sandwich structures, or winding techniques. These innovations could further mitigate deformation risks and improve structural efficiency.

## Conclusions

In conclusion, this study delved into the deflection characteristics of pGFRP cross-arms under normal and broken wire conditions, as well as the creep strain deflection in high transmission towers. The significant findings revealed several key points:

- The pGFRP cross-arm exhibited a linear correlation between load and deflection, behaving as a linear elastic material under both normal and broken wire conditions. Compared to Balau wood cross-arms, the pGFRP cross-arm displayed immediate response to load application and smaller deflections under working loads due to its superior fabrication properties, higher bending stress tolerance, toughness, and stiffness.
- The study demonstrated that pGFRP cross-arms showcased enhanced creep resistance over time compared to wood cross-arms. The Findley Power Law Model effectively characterized the creep behavior of both materials, with the pGFRP exhibiting lower transient creep and a unique material exponent outside the standard range, suggesting potential for structural enhancements.
- Finite Element Analysis (FEA) effectively predicted the deflection behavior of the pGFRP cross-arm, complementing experimental methods. The study highlighted the effectiveness of FEA in understanding the structural response of the composite material.
- The gaps identified in this research should be addressed in future studies, particularly the challenge of developing sustainable raw materials, such as those utilizing alkaline activation, to reduce the environmental impact of these structures, which was not explored.

The relationship between these findings underscores the importance of understanding the load-deflection behavior and creep characteristics of pGFRP cross-arms for structural design and performance evaluation. The study’s outcomes provide valuable insights for improving the mechanical performance of composite cross-arms in transmission towers. Others future research directions could focus on dynamic results, flexibility reactions, failure modes, and creep analyses under varied safety factors to enhance the understanding of these structures. The potential societal benefits of this research lie in the improved design and performance of pGFRP cross-arms, which are crucial components in high transmission towers. By enhancing the mechanical properties and creep resistance of these cross-arms, the study contributes to the reliability and safety of transmission infrastructure, benefiting society by ensuring the robustness and longevity of power transmission systems.

## Data Availability

The datasets used and analysed during the current study available from the corresponding author on reasonable request.
